# Supplementary material for: HDAC1: An Essential and Conserved Member of the Diverse Zn2+-Dependent HDAC Family Driven by Divergent Selection Pressure
Source: Int J Mol Sci. 2023 Dec 2;24(23):17072. doi: 10.3390/ijms242317072 (PMC10707265; doi:10.3390/ijms242317072)
Supplement: Supplementary file 1 [file ijms-24-17072-s001.zip › 20231030_s_end.pdf]

Supplementary Material

HDAC1: An Essential and Conserved Member of the Diverse Zn^{2+}-Dependent HDAC Family Driven by Divergent Selection Pressure

Jing-Fang Yang [1,2], Le-Rong Shi [1,2], Ke-Chen Wang [1,2], Li-Long Huang [1,2], Yun-Shuang Deng [1,2], Mo-Xian Chen [3], Fang-Hao Wan [1,4,*] and Zhong-Shi Zhou [1,2,*]

[1] State Key Laboratory for Biology of Plant Diseases and Insect Pests, Institute of Plant Protection, Chinese Academy of Agricultural Sciences, Beijing 100193, China; yangjingfang@caas.cn (J.-F.Y.); 82101222346@caas.cn (L.-R.S.); wangkechen1999@163.com (K.-C.W.); 15386163915@163.com (L.-L.H.); dys011128@163.com (Y.-S.D.)

[2] National Nanfan Research Institute (Sanya), Chinese Academy of Agricultural Sciences,
Sanya 572024, China

[3] National Key Laboratory of Green Pesticide, Key Laboratory of Green Pesticide and Agricultural Bioengineering, Ministry of Education, Guizhou University, Guiyang 550025, China; cmx2009920734@gmail.com

[4] Shenzhen Branch, Guangdong Laboratory for Lingnan Modern Agriculture, Genome Analysis Laboratory of the Ministry of Agriculture and Rural Affairs, Agricultural Genomics Institute at Shenzhen, Chinese Academy of Agricultural Sciences,
Shenzhen 518120, China

* Correspondence: wanfanghao@caas.cn (F.-H.W.); zhouzhongshi@caas.cn (Z.-S.Z.)

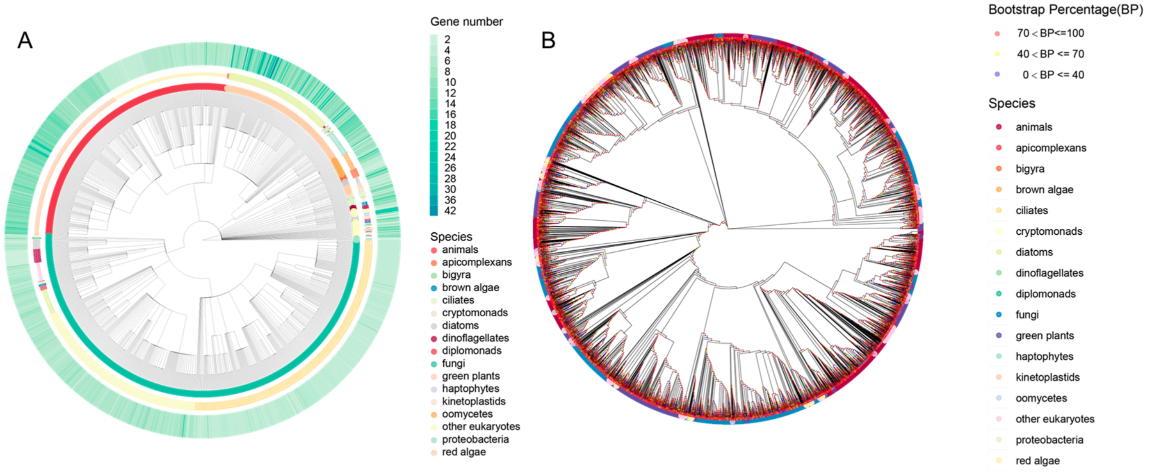

Figure S1. The repertoires and phylogenetic tree of HDACs from 1451 species. The statistical data for each class in these species is shown in Figure A. The corresponding detailed data is shown in Table S1 and Table S2. The common tree of the selected species was obtained from the NCBI database. These figures were prepared using the ggtree package in R programming. The Maximum Likelihood (ML) phylogenetic tree was built up using FastTree v1.4.3 (B).

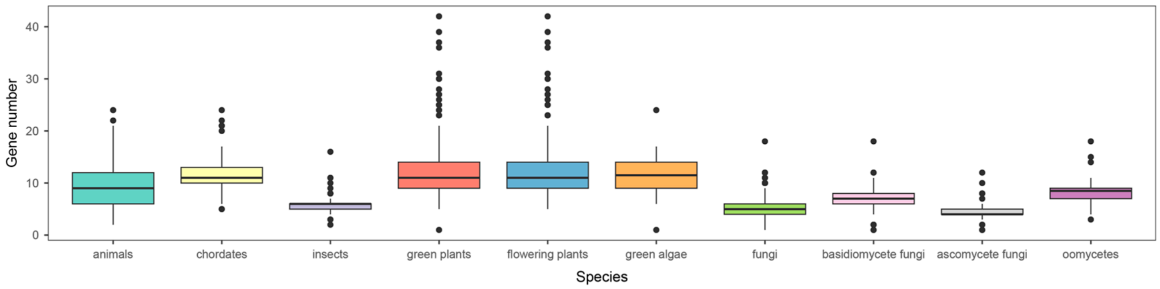

Figure S2. The boxplot for the *HDAC* gene numbers in the major groups of species.

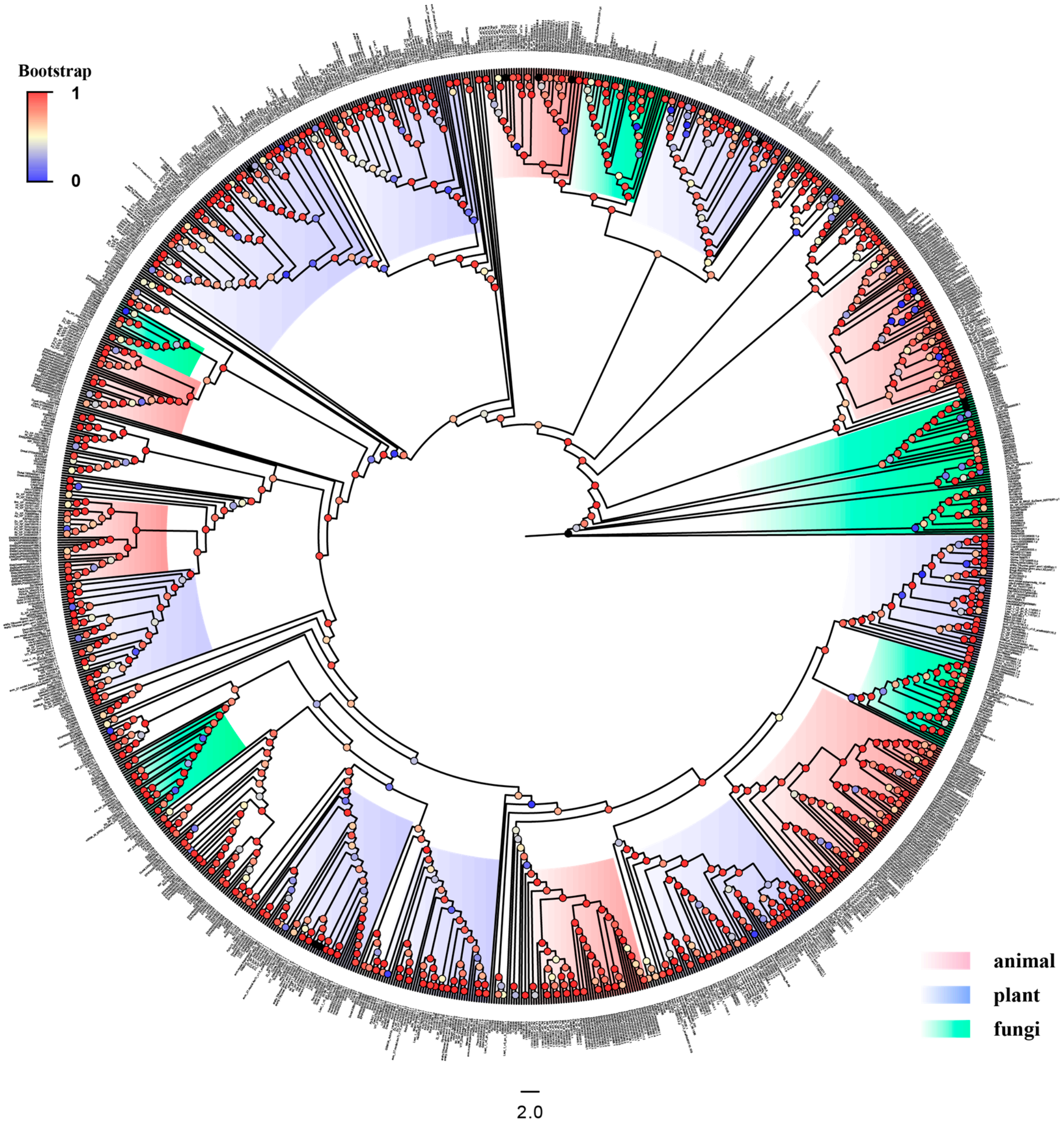

Figure S3. The phylogenetic tree of HDACs from the 142 selected species. The Maximum Likelihood (ML) phylogenetic tree was built up using FastTree v1.4.3. It was shown using FigTree v1.4.3.

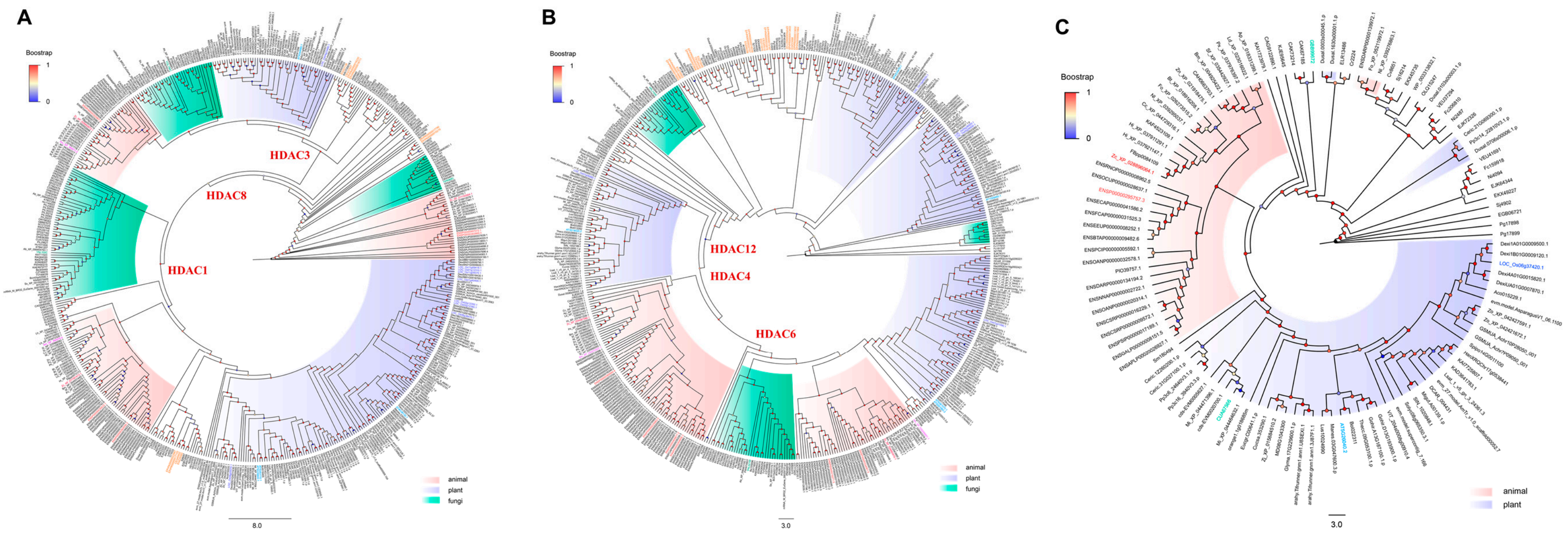

Figure S4. The phylogenetic trees of HDACs from the 142 selected species. These Maximum Likelihood (ML) phylogenetic trees were built up by PhyML v3.0 based on Q.pfam +R, VT +R, and Q.pfam +R models using the Class I, Class II, and Class IV sequences, respectively. The taxa of *H. sapiens*, *A. mellifera*, *S. cerevisiae*, *P. sojae*, *A. thaliana*, and *O. sativa* are colored red, pink, green, orange, cyan, and slate, respectively. They were prepared through FigTree v1.4.3.

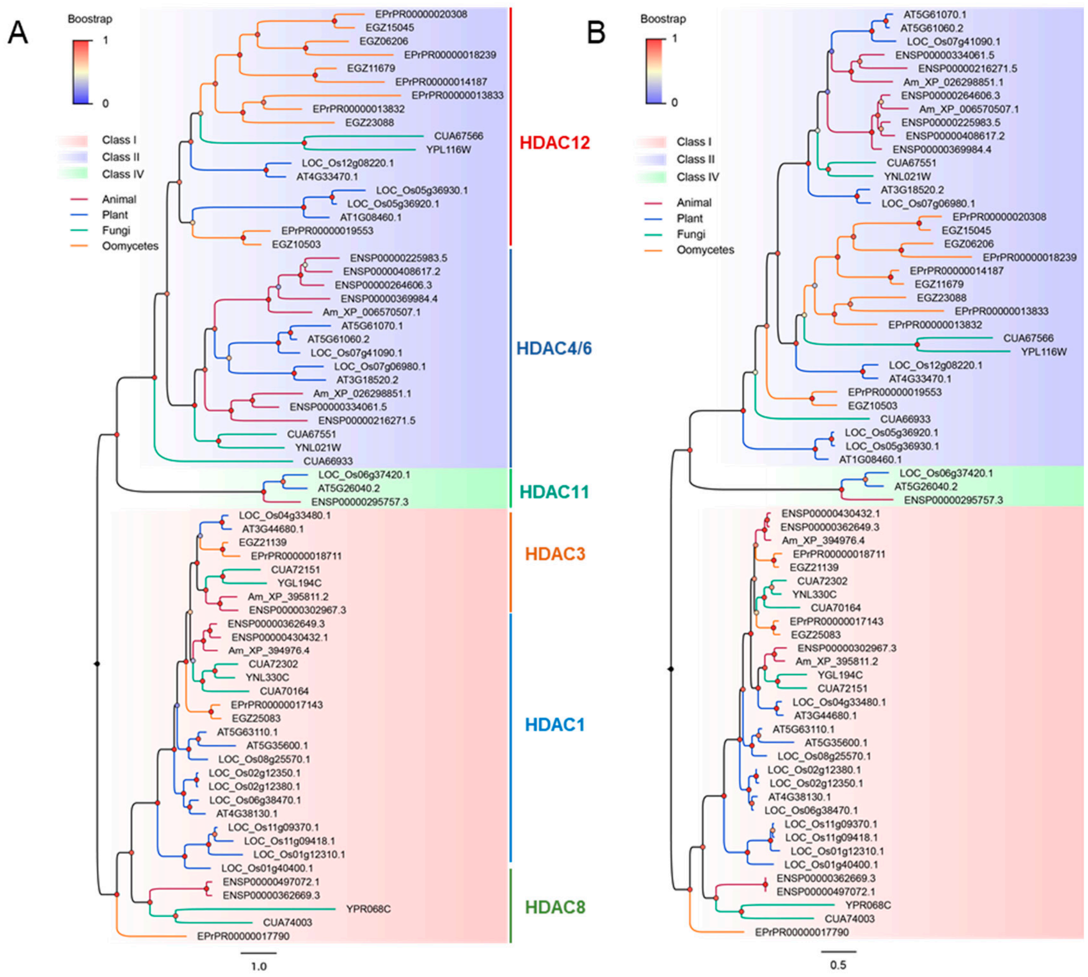

Figure S5. The phylogenetic trees of HDACs from the eight representative species. These Maximum Likelihood (ML) phylogenetic trees were built up by PhyML v3.0 based on VT +R (A) and LG +R (B) models using the whole sequences (A) and the Hist_deacetyl domain sequences (B), respectively. They were prepared through FigTree v1.4.3.

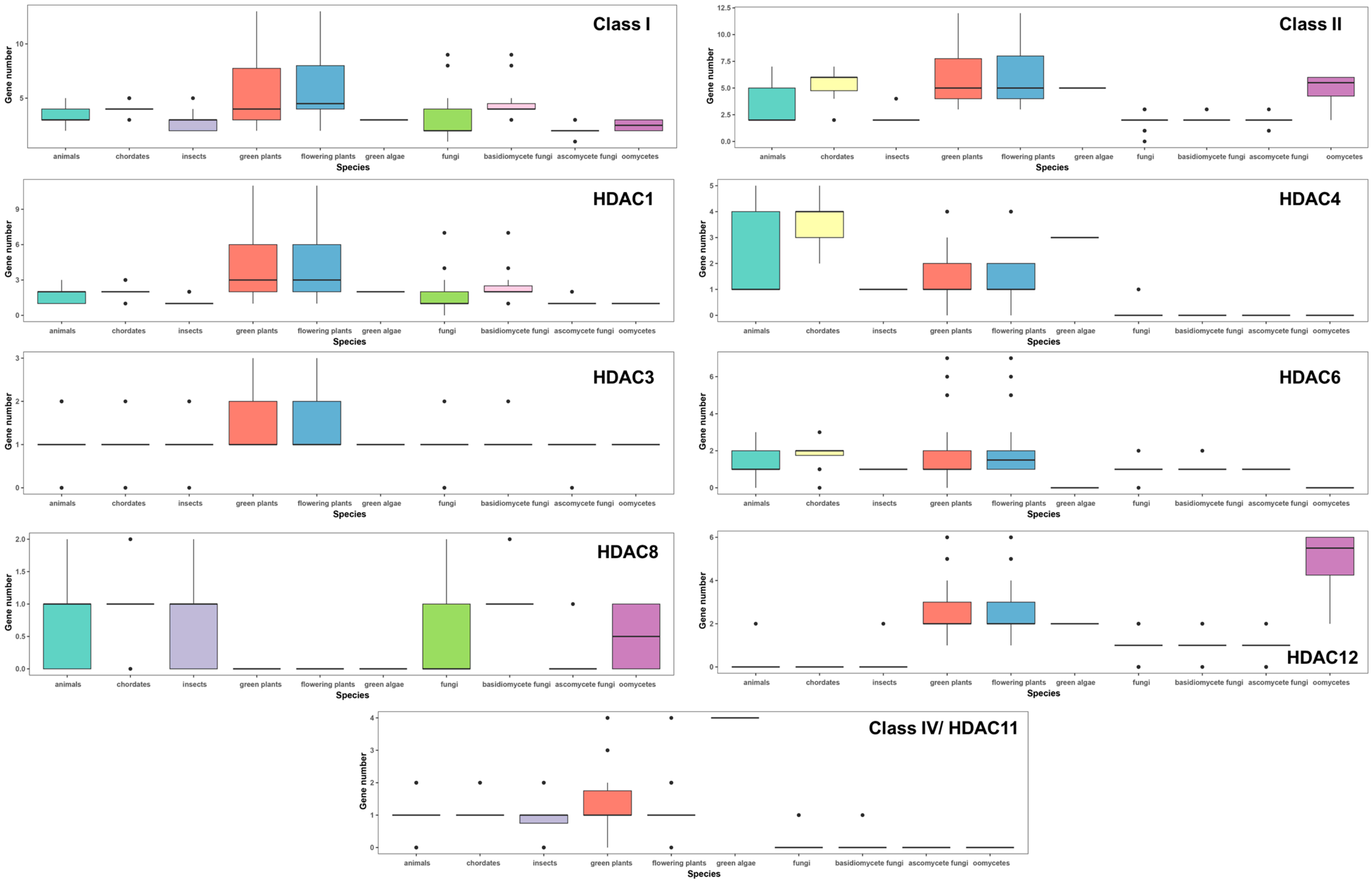

Figure S6. The boxplots for the different types of *HDAC* gene numbers in the major groups of species.

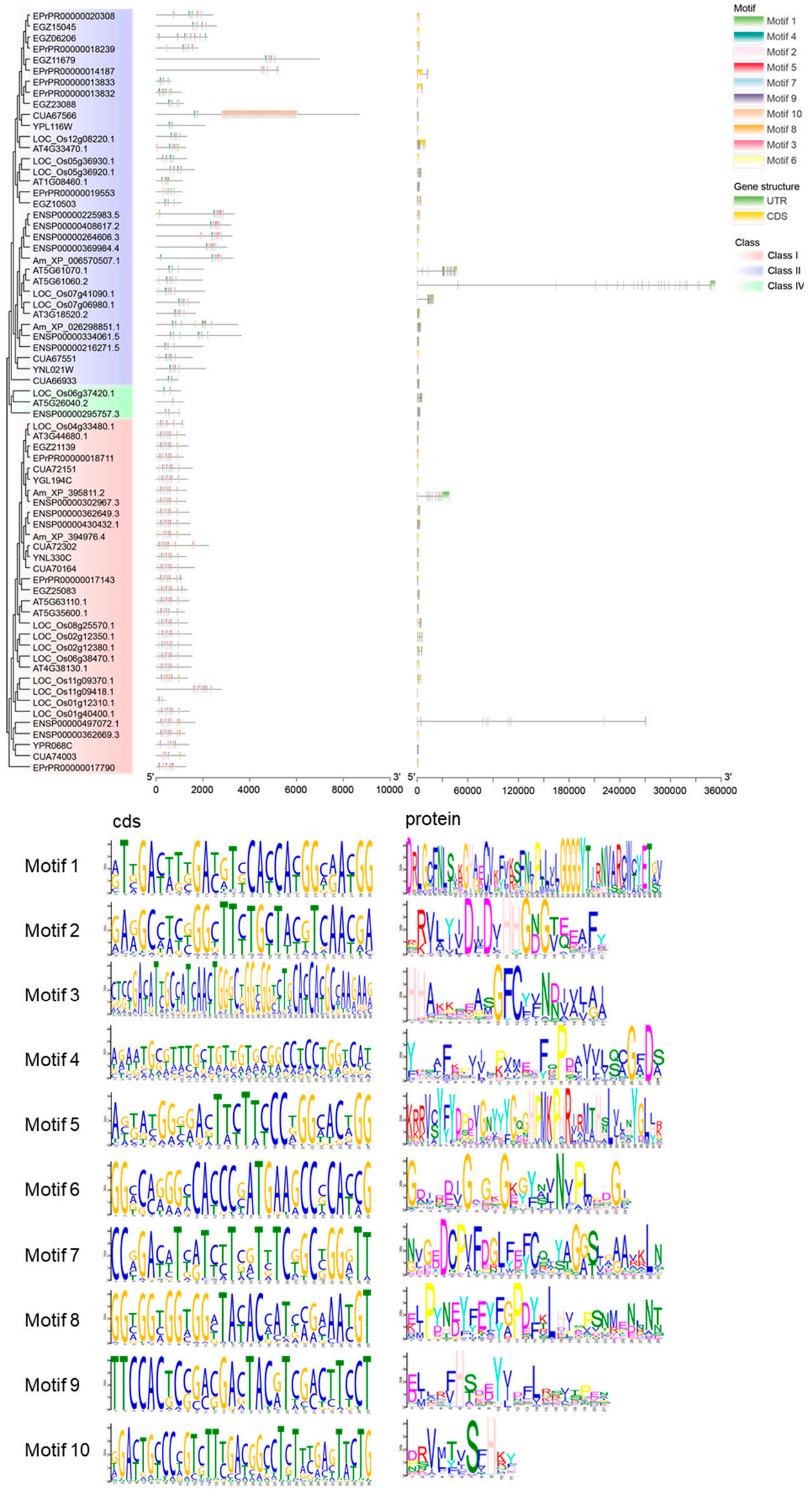

Figure S7. The cds motifs and gene structures of sequences. The motifs were annotated using the TBtools v1.120, and the gene structures were extracted from the database. The cds and protein motifs are shown.

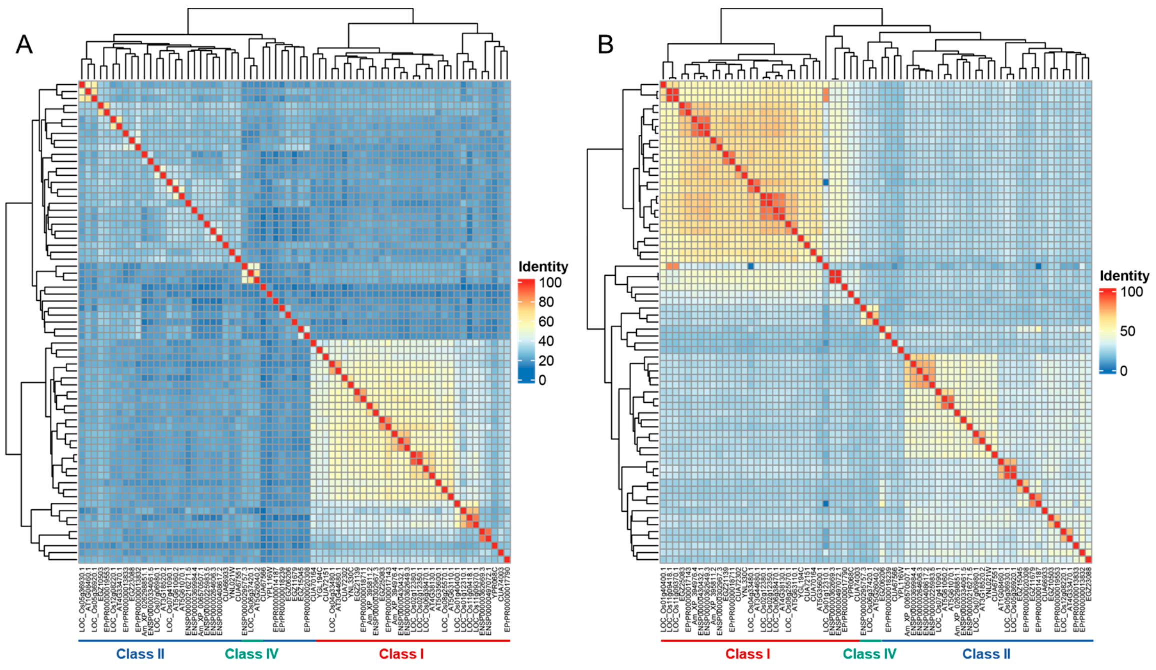

Figure S8. The heatmaps of sequences identity of HDACs from the eight representative species. The heatmaps of whole sequences identity (A) and the Hist_deacetyl domain sequences identity (B) were generated using the pheatmap package in R programming.

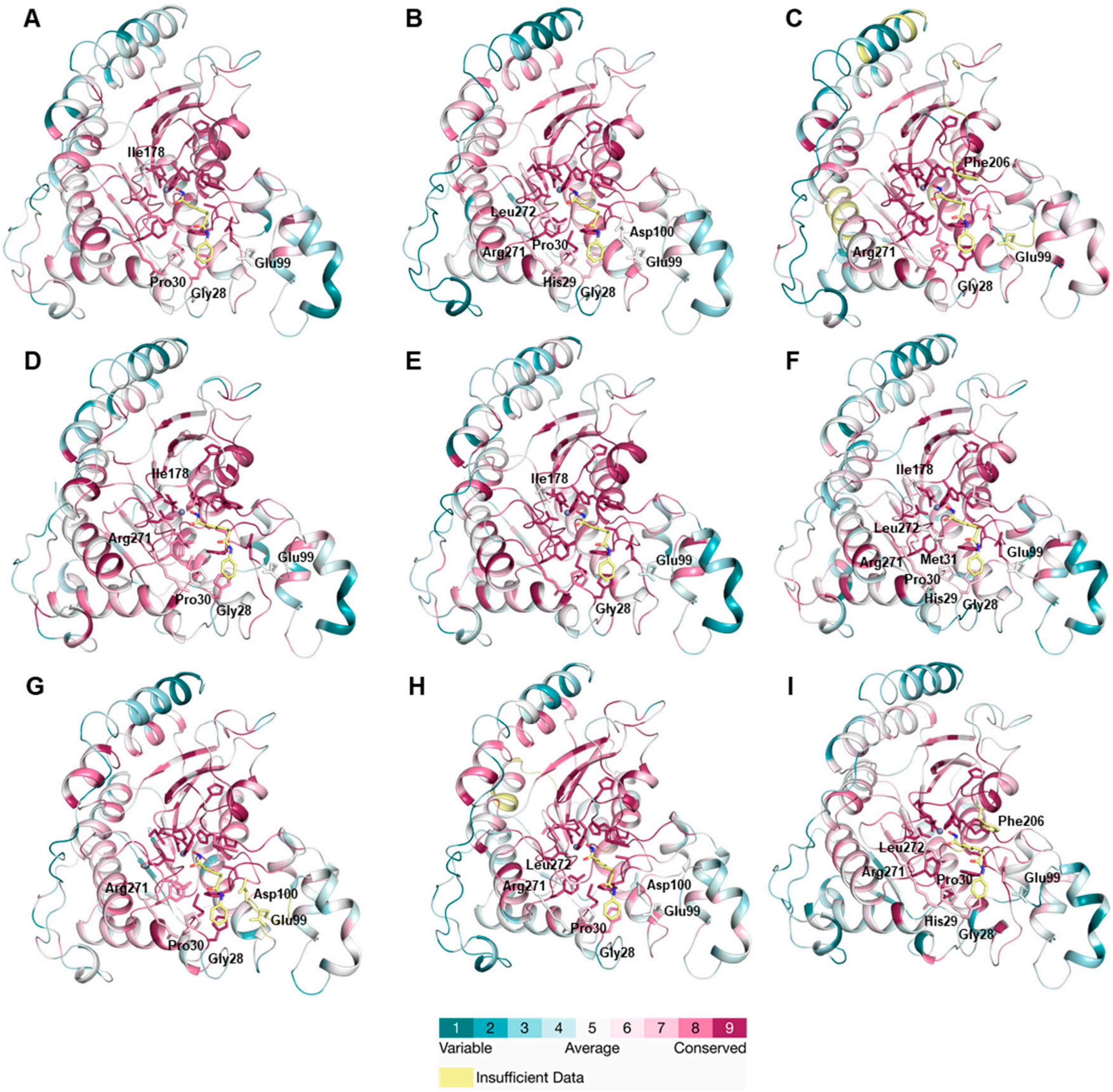

Figure S9. The 3D structure of human HDAC2 showing sequence conservation. The conservations (ConSurf grades) of residues were calculated using Class I (A), Class II (B), Class IV (C), HDAC1 (D), HDAC3 (E), HDAC8 (F), HDAC4 (G), HDAC6 (H), and HDAC12 (I) sequences from 1451 species. The human HDAC2 crystal structures complex with vorinostat (PDBID: 4lxz) are colored according to ConSurf grade from blue (low grade) to red (high grade). The complexed vorinostat and residues contacting the Zn atom and vorinostat are shown in sticks. The residues within the 6 Å distance to vorinostat with low ConSurf grade (≤7) are also shown in sticks.

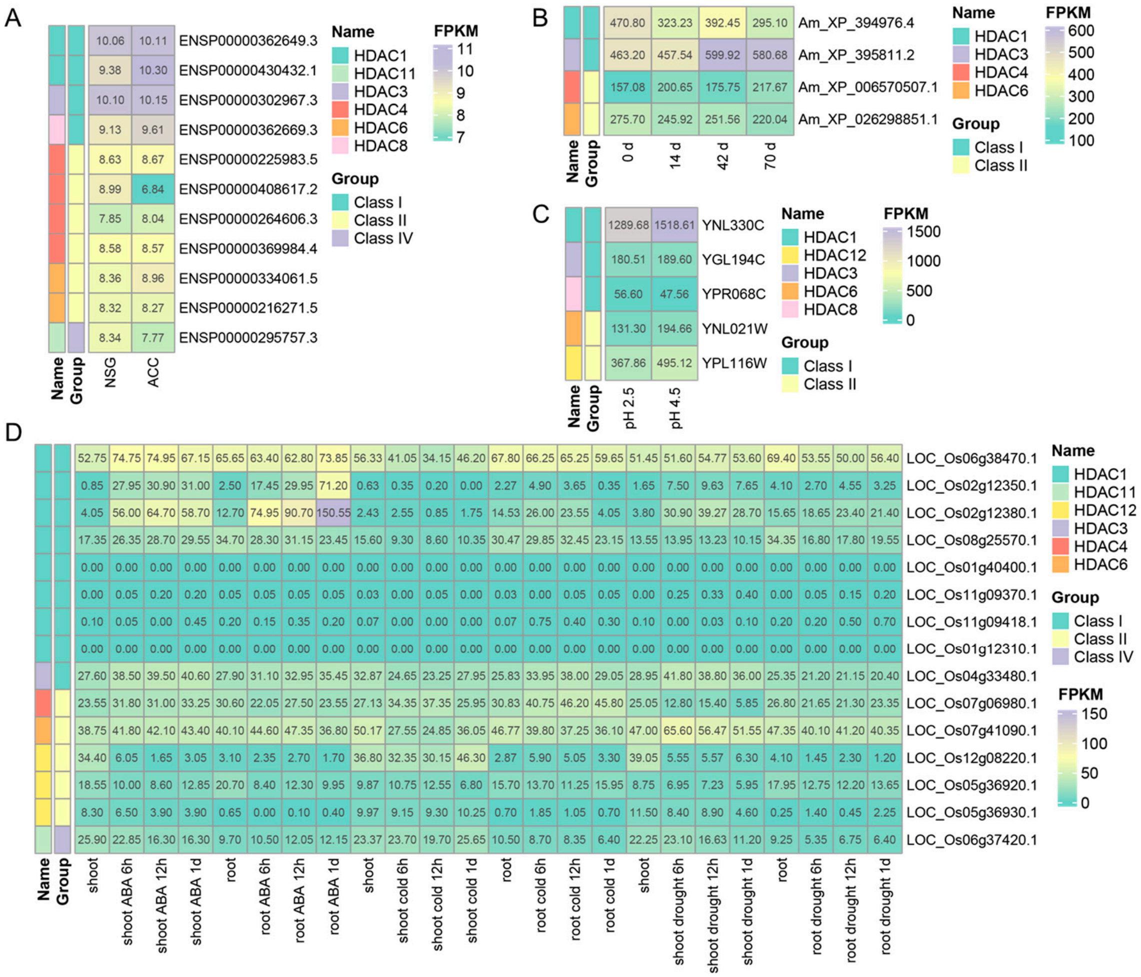

Figure S10. The gene expressions of HDACs in *H. sapiens*, *A. mellifera*, *S. cerevisiae*, and *O. sativa*. The data in normal salivary gland tissue (NSG)/adenoid cystic carcinoma (ACC) of *H. sapiens*, in *A. mellifera* installed into eggplant greenhouses after 0 days, 14 days, 42 days, and 70 days, in *S. cerevisiae* treated with pH 2.5 and pH 4.5, and in *O. sativa* under ABA, cold, and drought treatment in different tissues are shown with heatmap.

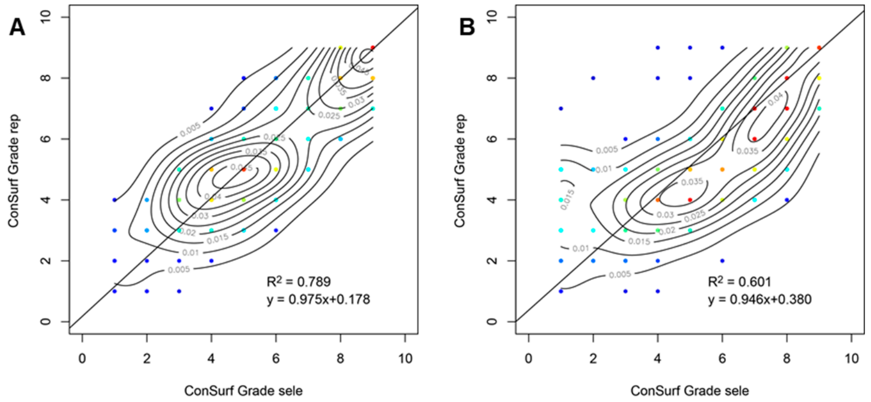

Figure S11. The correlation of sequence conservation of the eight representative species and the 142 selected species. The conservations (ConSurf grades) of residues were calculated using Class I (A) and Class II (B) sequences from the 142 selected species (x-axis) and the eight representative species (y-axis). The points are colored based on the densities from blue (low) to red (high).

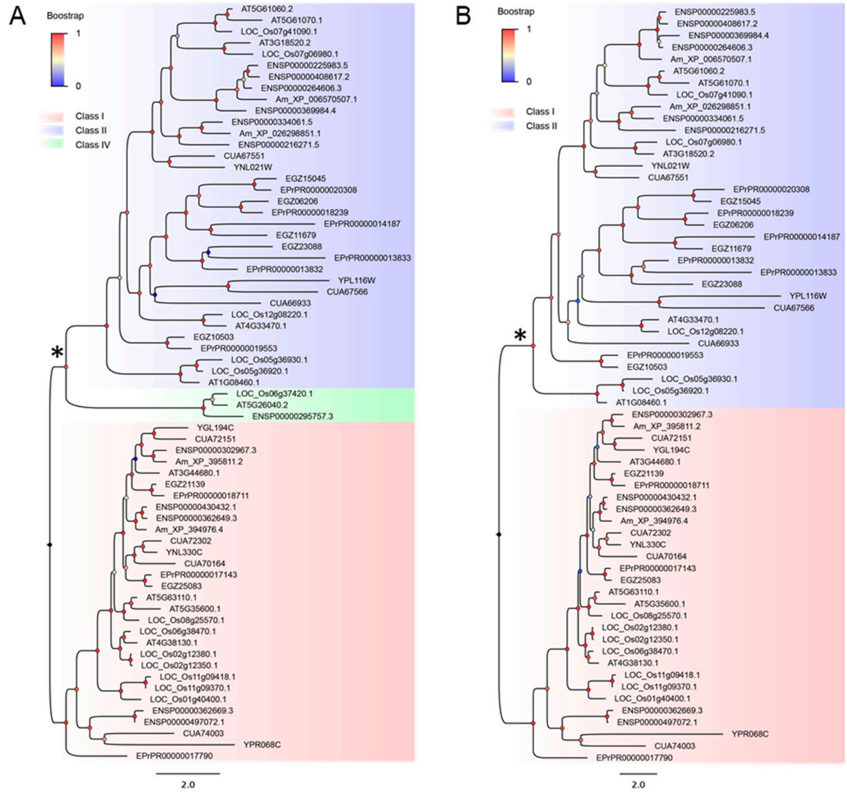

Figure S12. The phylogenetic trees of HDACs used for the positive selection (PS) and functional divergence (FD) analysis. These Maximum Likelihood (ML) phylogenetic

trees were built up by PhyML v3.0 based on VT +R and Q.pfam +R models using the sequences of Class I, II, and IV and Class I and Class II, respectively. The short sequences (LOC_Os04g33480.1 and LOC_Os01g12310.1) were deleted in this work. The nodes for two ω values in Two-ratio model calculations are labeled using black. The cds sequences of proteins were used for further PS and FD analysis (A). These trees were prepared through FigTree v1.4.3.

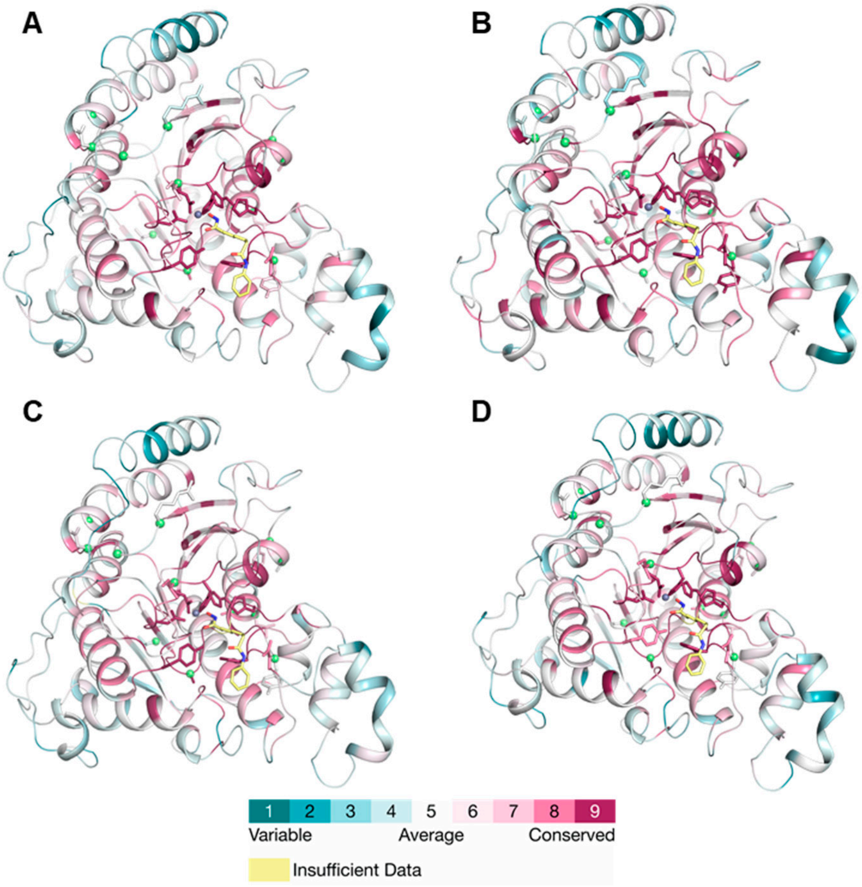

Figure S13. The 3D structure of human HDAC2 showing sequence conservation and functional divergence (FD) I sites. The conservations (ConSurf grades) of residues were calculated using all (A), Class I (B), Class II (C), and Class II and Class IV (D) sequences of eight representative species. The CA atoms of residues involved in FD among Class I and Class II are shown as green balls. The human HDAC2 crystal structures complex with vorinostat (PDBID: 4lxz) are colored according to ConSurf grade from blue (low grade) to red (high grade). The complexed vorinostat and important residues are shown in sticks.

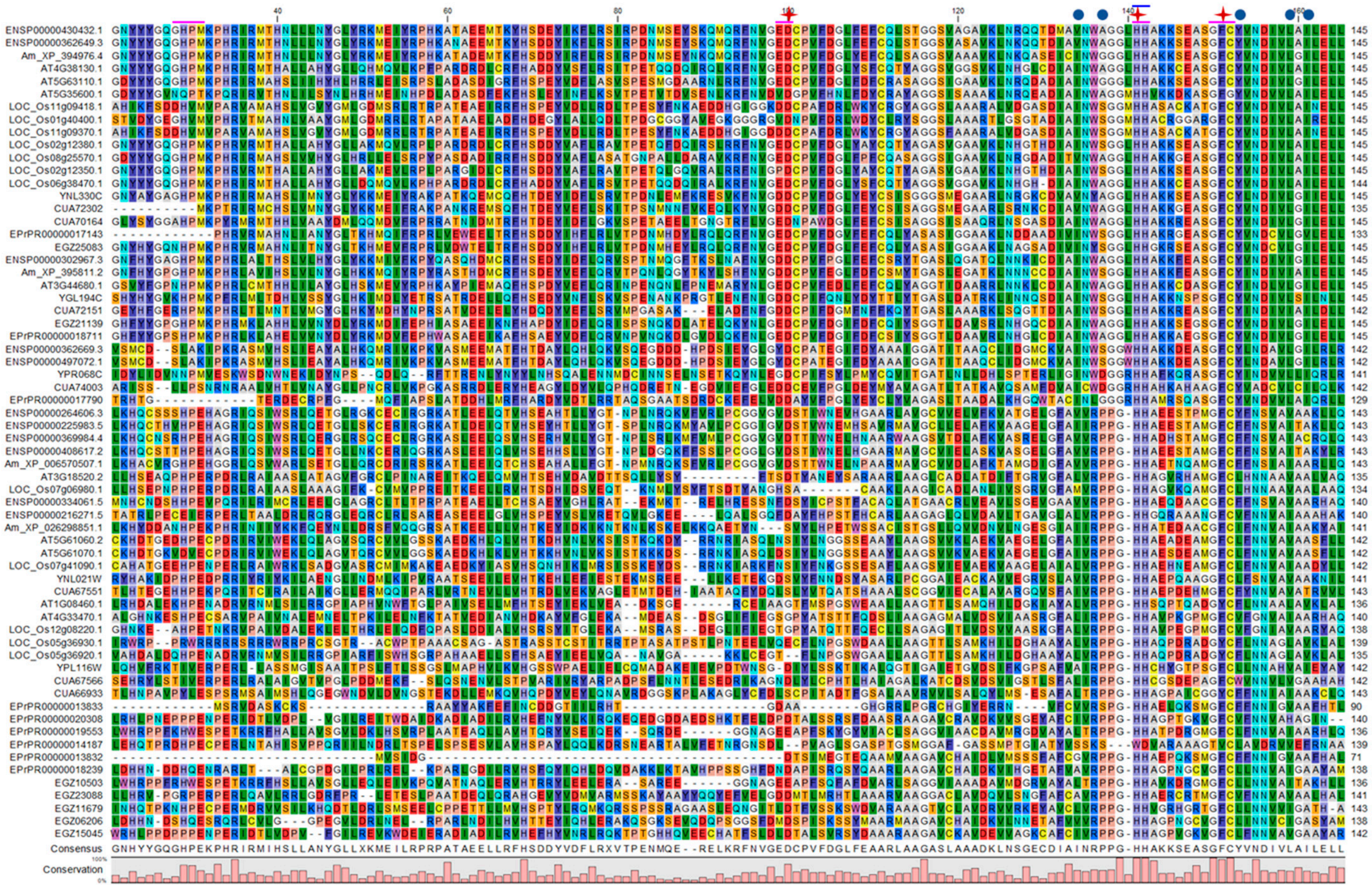

ENSP00000430432.1
ENSP00000362649.3
Am_XP_394976.4
AT4G38130.1
AT5G63110.1
AT5G35600.1
LOC_Os11g09418.1
LOC_Os01g40400.1
LOC_Os11g09370.1
LOC_Os02g12380.1
LOC_Os08g25570.1
LOC_Os02g12350.1
LOC_Os06g38470.1
YNL330C
CUA72302
CUA70164
EPrPR00000017143
EGZ25083
ENSP00000302967.3
Am_XP_395811.2
AT3G44680.1
YGL194C
CUA72151
EGZ21139
EPrPR00000018711
ENSP00000362669.3
ENSP00000497072.1
YPR068C
CUA74003
EPrPR00000017790
ENSP00000264606.3
ENSP00000225983.5
ENSP00000369984.4
ENSP00000408617.2
Am_XP_006570507.1
AT3G18520.2
LOC_Os07g06980.1
ENSP00000334061.5
ENSP00000216271.5
Am_XP_026298851.1
AT5G61060.2
AT5G61070.1
LOC_Os07g41090.1
YNL021W
CUA67551
AT1G08460.1
AT4G33470.1
LOC_Os12g08220.1
LOC_Os05g36930.1
LOC_Os05g36920.1
YPL116W
CUA67566
CUA66933
EPrPR00000013833
EPrPR00000020308
EPrPR00000019553
EPrPR00000014187
EPrPR00000013832
EPrPR00000018239
EGZ10503
EGZ23088
EGZ11679
EGZ06206
EGZ15045
Consensus
Conservation

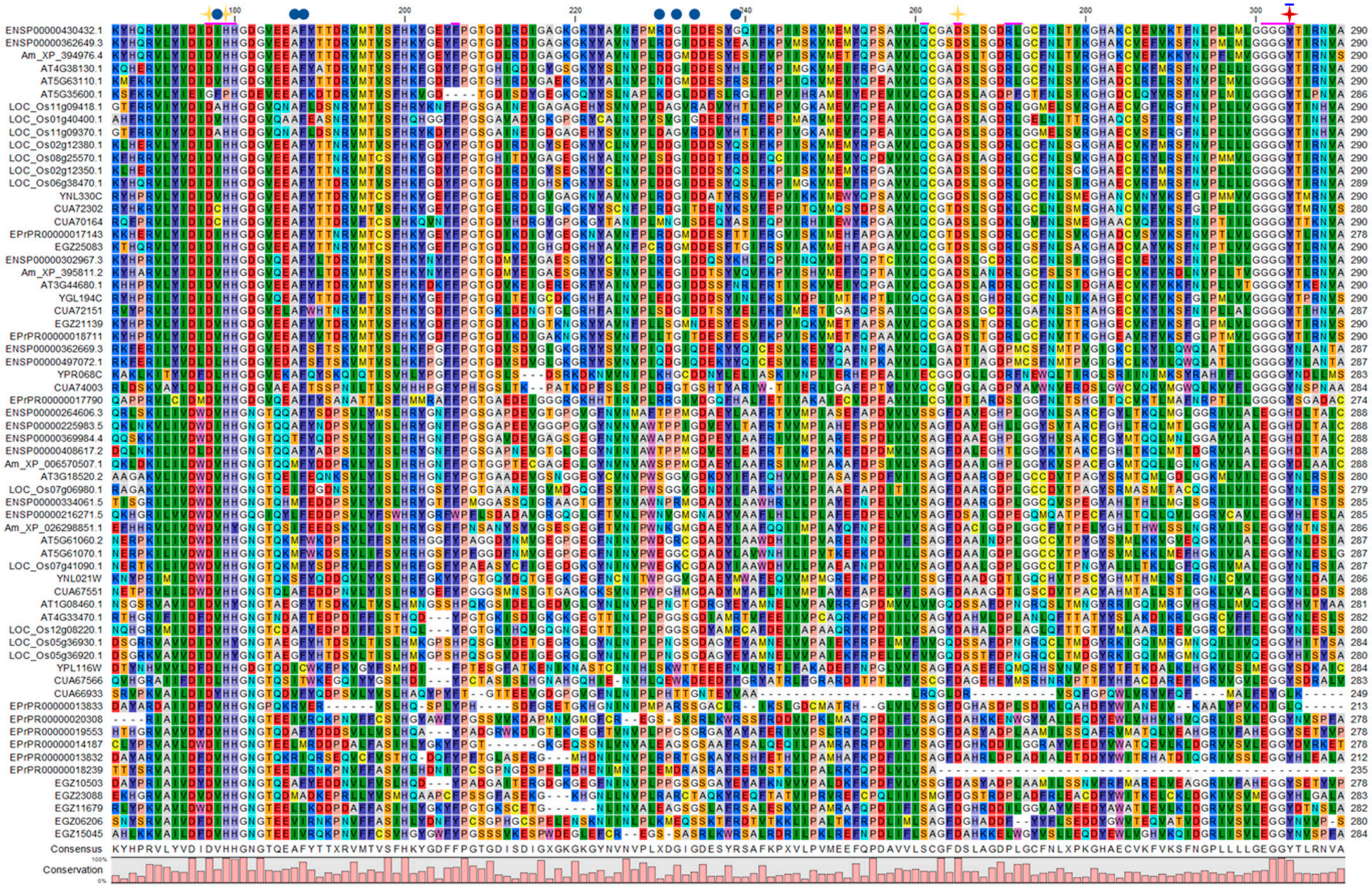

Figure S14. Sequence alignments of HDACs in Class I and Class II from eight selected representative species with the ENSP00000430432.1 (HDAC2) of *H. sapiens*. The amino acids within the 6 Å distance to the vorinostat are marked with pink lines. The residues coordinating with the Zn atom and contacting with vorinostat are marked with orange stars and red stars. The catalytic amino acids are marked with blue lines. The residues under FD are marked with blue circles.

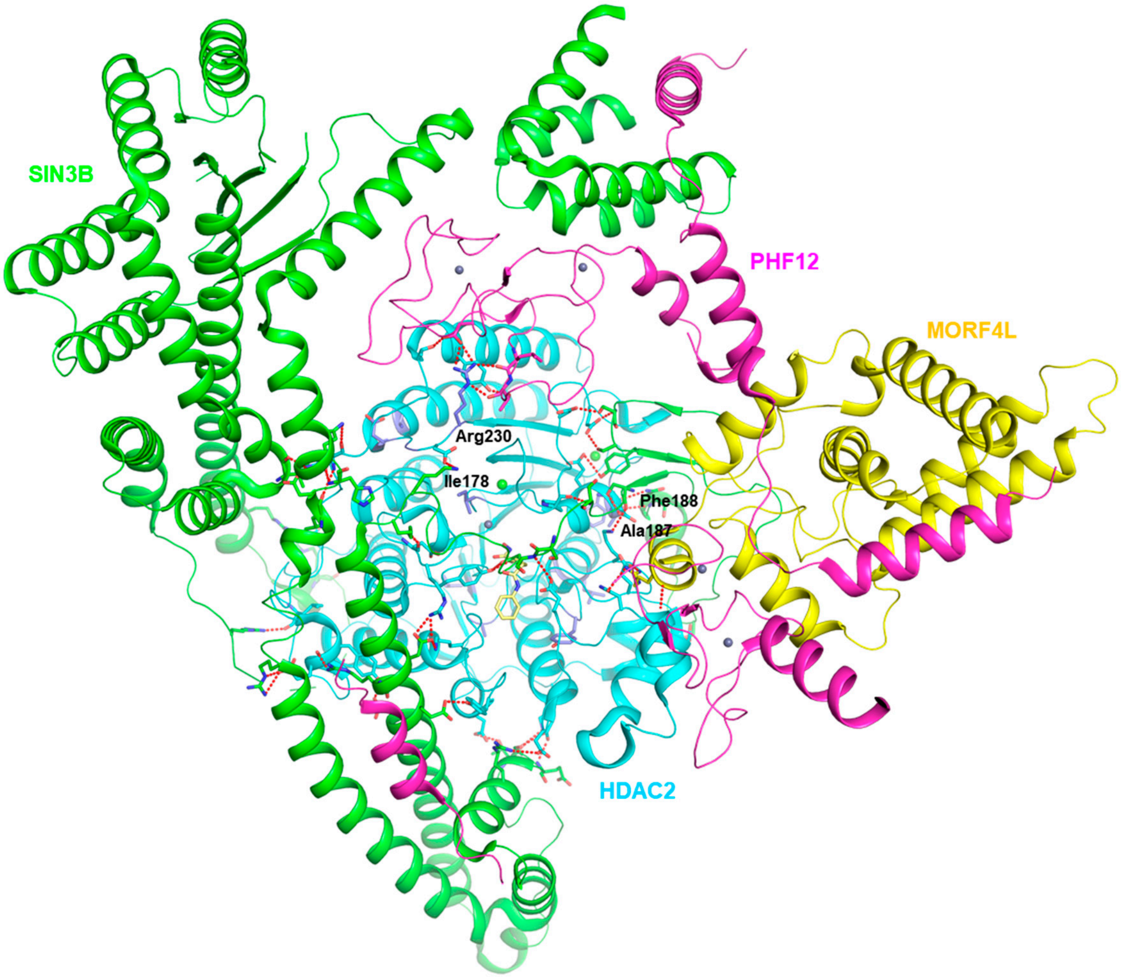

Figure S15. The crystal structure of the human SIN3B HDAC2 complex. This 3D structure (PDBID: 8c60) is downloaded from the PDB database. Chains are shown as cartoons. The chains of HDAC2, paired amphipathic helix protein Sin3b (SIN3B), PHD finger protein 12 (PHF12), and mortality factor 4-like protein 1 (MORF4L) are colored in cyan, green, magenta, and yellow, respectively. The residues responsible for FD I are shown in sticks and colored in slate. The critical residues among them are labeled with residue names. The hydrogen bonds are shown as red dashes.

Table S1. The HDAC repertoires of 1451 species were determined in this study.

Tax name	Gene	Protein	Subphyla	Species
Acanthamoeba castellanii str. Neff	7	7	other eukaryotes	other eukaryotes
Vitrella brassicaformis CCMP3155	12	12	other eukaryotes	other eukaryotes
Chromera velia CCMP2878	9	9	chromeraceae	other eukaryotes
Bigelowiella natans CCMP2755	12	12	cercozoans	other eukaryotes
Fonticula alba	7	8	rotosphaerida	other eukaryotes
Sphaeroforma arctica JP610	8	8	ichthyosporea	other eukaryotes
Capsaspora owczarzaki ATCC 30864	6	8	filasterea	other eukaryotes
Ochromonadaceae sp. CCMP2298	14	14	synurophyceae	other eukaryotes
Thecamonas trahens ATCC 50062	5	5	apusomonadida	other eukaryotes
Salpingoeca rosetta	4	4	choanoflagellates	other eukaryotes
Monosiga brevicollis MX1	2	2	choanoflagellates	other eukaryotes
Perkinsus marinus ATCC 50983	12	15	perkinsozoa	other eukaryotes
Tritrichomonas foetus	5	5	parabasalia	other eukaryotes
Trichomonas vaginalis G3	9	9	trichomonads	other eukaryotes
Tribonema minus	15	15	yellow-green algae	other eukaryotes
Nannochloropsis gaditana	10	10	eustigmatophyceae	other eukaryotes
Reticulomyxa filosa	11	11	forams	other eukaryotes
Pelagophyceae sp. CCMP2097	11	11	pelagophytes	other eukaryotes
Aureococcus anophagefferens	7	7	pelagophytes	other eukaryotes
Hyphochytrium catenoides	16	16	hyphochytriomycetes	other eukaryotes
Ochromonas sp. CCMP1393	16	16	golden algae	other eukaryotes
Paraphysomonas imperforata	9	9	golden algae	other eukaryotes
Plasmodiophora brassicae	4	4	endomyxa	other eukaryotes
Pavlovales sp. CCMP2436	10	10	haptophytes	haptophytes
Emiliania huxleyi CCMP1516	27	27	haptophytes	haptophytes
Chrysochromulina parva	18	18	haptophytes	haptophytes
Phaeocystis globosa	18	18	haptophytes	haptophytes
Phaeocystis antarctica	23	23	haptophytes	haptophytes
Fistulifera solaris	18	18	diatoms	diatoms
Seminavis robusta	14	14	diatoms	diatoms
Phaeodactylum tricornutum CCAP 1055/1	12	12	diatoms	diatoms
Nitzschia inconspicua	8	8	diatoms	diatoms
Fragilariopsis cylindrus	10	10	diatoms	diatoms
Pseudo-nitzschia multistriata	8	8	diatoms	diatoms
Minidiscus variabilis	13	13	diatoms	diatoms
Thalassiosira oceanica	8	8	diatoms	diatoms
Cyclotella cryptica	11	11	diatoms	diatoms
Labyrinthula sp.	6	6	labyrinthulida	bigyra
Hondaea fermentalgiana	12	12	thraustochytrida	bigyra
Schizochytrium aggregatum ATCC 28209	12	12	thraustochytrida	bigyra
Aurantiochytrium limacinum ATCC MYA-1381	12	12	thraustochytrida	bigyra

Aplanochytrium kerguelense PBS07	13	13	thraustochytrida	bigyra
Cafeteria roenbergensis	6	6	bicosoecida	bigyra
Blastocystis hominis	4	4	opalinata	bigyra
Erpetoichthys calabaricus	11	18	chordates	animals
Pteropus vampyrus	11	11	chordates	animals
Rhinolophus ferrumequinum	10	30	chordates	animals
Myotis lucifugus	14	14	chordates	animals
Tupaia belangeri	9	9	chordates	animals
Monodelphis domestica	9	20	chordates	animals
Nannospalax galili	11	15	chordates	animals
Jaculus jaculus	13	26	chordates	animals
Chinchilla lanigera	11	27	chordates	animals
Castor canadensis	11	17	chordates	animals
Fukomys damarensis	11	15	chordates	animals
Heterocephalus glaber	16	28	chordates	animals
Octodon degus	13	22	chordates	animals
Cavia aperea	8	20	chordates	animals
Cavia porcellus	11	19	chordates	animals
Rattus norvegicus	13	16	chordates	animals
Mus musculus	11	53	chordates	animals
Meriones unguiculatus	10	23	chordates	animals
Peromyscus maniculatus bairdii	12	18	chordates	animals
Microtus ochrogaster	12	21	chordates	animals
Mesocricetus auratus	12	26	chordates	animals
Cricetulus griseus	14	28	chordates	animals
Dipodomys ordii	11	22	chordates	animals
Spermophilus dauricus	12	14	chordates	animals
Sciurus vulgaris	11	27	chordates	animals
Ictidomys tridecemlineatus	11	19	chordates	animals
Urocitellus parryii	10	21	chordates	animals
Marmota marmota marmota	12	18	chordates	animals
Oryctolagus cuniculus	10	48	chordates	animals
Ochotona princeps	9	9	chordates	animals
Procavia capensis	10	10	chordates	animals
Equus caballus	11	58	chordates	animals
Loxodonta africana	11	20	chordates	animals
Cervus hanglu yarkandensis	14	47	chordates	animals
Moschus moschiferus	16	34	chordates	animals
Catagonus wagneri	11	25	chordates	animals
Phocoena sinus	13	36	chordates	animals
Bison bison bison	8	14	chordates	animals
Ovis aries	11	12	chordates	animals
Capra hircus	12	28	chordates	animals
Bos taurus	11	34	chordates	animals
Vicugna pacos	5	5	chordates	animals
Camelus dromedarius	11	33	chordates	animals

Sus scrofa	11	58	chordates	animals
Balaenoptera musculus	12	34	chordates	animals
Physeter catodon	12	37	chordates	animals
Monodon monoceros	12	30	chordates	animals
Delphinapterus leucas	13	39	chordates	animals
Tursiops truncatus	11	11	chordates	animals
Suricata suricatta	11	45	chordates	animals
Zalophus californianus	12	49	chordates	animals
Lynx canadensis	11	43	chordates	animals
Panthera leo	10	31	chordates	animals
Felis catus	11	42	chordates	animals
Neogale vison	12	35	chordates	animals
Mustela putorius furo	11	11	chordates	animals
Ailuropoda melanoleuca	10	11	chordates	animals
Ursus maritimus	9	22	chordates	animals
Vulpes vulpes	11	47	chordates	animals
Canis lupus familiaris	10	33	chordates	animals
Carlito syrichta	12	24	chordates	animals
Prolemur simus	12	36	chordates	animals
Propithecus coquereli	10	33	chordates	animals
Nomascus leucogenys	11	33	chordates	animals
Aotus nancymaae	10	43	chordates	animals
Otolemur garnettii	10	10	chordates	animals
Microcebus murinus	11	37	chordates	animals
Homo sapiens	12	170	chordates	animals
Pongo abelii	12	13	chordates	animals
Pan paniscus	11	44	chordates	animals
Gorilla gorilla gorilla	11	46	chordates	animals
Piliocolobus tephrosceles	17	50	chordates	animals
Colobus angolensis palliatus	14	48	chordates	animals
Rhinopithecus bieti	16	47	chordates	animals
Chlorocebus sabaeus	13	13	chordates	animals
Mandrillus leucophaeus	12	38	chordates	animals
Theropithecus gelada	14	45	chordates	animals
Papio anubis	14	47	chordates	animals
Macaca fascicularis	15	50	chordates	animals
Cercocebus atys	12	48	chordates	animals
Cebus imitator	14	42	chordates	animals
Saimiri boliviensis boliviensis	15	50	chordates	animals
Callithrix jacchus	13	52	chordates	animals
Echinops telfairi	10	10	chordates	animals
Sorex araneus	6	6	chordates	animals
Erinaceus europaeus	10	10	chordates	animals
Dasypus novemcinctus	10	15	chordates	animals
Choloepus hoffmanni	6	6	chordates	animals
Phascolarctos cinereus	11	28	chordates	animals

Vombatus ursinus	12	16	chordates	animals
Notamacropus eugenii	10	10	chordates	animals
Sarcophilus harrisii	12	12	chordates	animals
Ornithorhynchus anatinus	10	10	chordates	animals
Struthio camelus australis	9	26	chordates	animals
Buteo japonicus	9	15	chordates	animals
Aquila chrysaetos chrysaetos	10	15	chordates	animals
Accipiter nisus	11	21	chordates	animals
Calidris pugnax	10	37	chordates	animals
Falco tinnunculus	9	24	chordates	animals
Nothoprocta perdicaria	9	12	chordates	animals
Strix occidentalis caurina	10	17	chordates	animals
Otus sunia	8	14	chordates	animals
Athene cunicularia	9	13	chordates	animals
Bubo bubo	9	14	chordates	animals
Strigops habroptila	11	27	chordates	animals
Amazona collaria	9	15	chordates	animals
Melopsittacus undulatus	10	17	chordates	animals
Malurus cyaneus samueli	8	11	chordates	animals
Zosterops lateralis melanops	8	15	chordates	animals
Corvus moneduloides	11	27	chordates	animals
Manacus vitellinus	9	17	chordates	animals
Lepidothrix coronata	9	11	chordates	animals
Cyanoderma ruficeps	7	10	chordates	animals
Catharus ustulatus	10	22	chordates	animals
Ficedula albicollis	9	9	chordates	animals
Lonchura striata domestica	8	14	chordates	animals
Taeniopygia guttata	12	27	chordates	animals
Camarhynchus parvulus	10	16	chordates	animals
Geospiza fortis	10	13	chordates	animals
Chloebia gouldiae	11	19	chordates	animals
Zonotrichia albicollis	7	23	chordates	animals
Junco hyemalis	11	21	chordates	animals
Cyanistes caeruleus	10	16	chordates	animals
Parus major	10	26	chordates	animals
Serinus canaria	9	11	chordates	animals
Coturnix japonica	9	19	chordates	animals
Chrysolophus pictus	8	20	chordates	animals
Phasianus colchicus	9	17	chordates	animals
Pavo cristatus	9	28	chordates	animals
Gallus gallus	10	16	chordates	animals
Numida meleagris	9	18	chordates	animals
Cairina moschata domestica	10	19	chordates	animals
Anser cygnoides	7	16	chordates	animals
Anas platyrhynchos	9	26	chordates	animals
Apteryx haastii	9	15	chordates	animals

Dromaius novaehollandiae	10	18	chordates	animals
Pogona vitticeps	11	31	chordates	animals
Salvator merianae	11	21	chordates	animals
Podarcis muralis	11	36	chordates	animals
Varanus komodoensis	11	25	chordates	animals
Anolis carolinensis	11	11	chordates	animals
Naja naja	11	26	chordates	animals
Pseudonaja textilis	11	23	chordates	animals
Notechis scutatus	11	21	chordates	animals
Laticauda laticaudata	10	17	chordates	animals
Sphenodon punctatus	11	13	chordates	animals
Crocodylus porosus	8	24	chordates	animals
Pelusios castaneus	11	37	chordates	animals
Chelonoidis abingdonii	11	30	chordates	animals
Gopherus agassizii	11	36	chordates	animals
Pelodiscus sinensis	10	12	chordates	animals
Terrapene carolina triunguis	9	25	chordates	animals
Chrysemys picta bellii	11	34	chordates	animals
Chelydra serpentina	12	21	chordates	animals
Leptobrachium leishanense	11	28	chordates	animals
Lithobates catesbeianus	6	10	chordates	animals
Xenopus tropicalis	9	67	chordates	animals
Myripristis murdjan	13	55	chordates	animals
Sphaeramia orbicularis	14	64	chordates	animals
Larimichthys crocea	14	74	chordates	animals
Parambassis ranga	13	76	chordates	animals
Gouania willdenowi	13	55	chordates	animals
Salarias fasciatus	12	55	chordates	animals
Paramormyrops kingsleyae	13	25	chordates	animals
Scleropages formosus	12	80	chordates	animals
Hippocampus comes	13	15	chordates	animals
Stegastes partitus	14	27	chordates	animals
Amphiprion ocellaris	15	26	chordates	animals
Acanthochromis polyacanthus	13	22	chordates	animals
Betta splendens	13	70	chordates	animals
Anabas testudineus	13	23	chordates	animals
Labrus bergylta	15	27	chordates	animals
Cynoglossus semilaevis	12	24	chordates	animals
Scophthalmus maximus	14	26	chordates	animals
Periophthalmus magnuspinnatus	11	18	chordates	animals
Neogobius melanostomus	9	24	chordates	animals
Mastacembelus armatus	14	30	chordates	animals
Monopterus albus	13	25	chordates	animals
Echeneis naucrates	12	71	chordates	animals
Seriola dumerili	14	20	chordates	animals
Mola mola	13	14	chordates	animals

Tetraodon nigroviridis	13	16	chordates	animals
Takifugu rubripes	13	59	chordates	animals
Dicentrarchus labrax	13	71	chordates	animals
Lates calcarifer	16	66	chordates	animals
Sparus aurata	14	80	chordates	animals
Pundamilia nyererei	14	20	chordates	animals
Maylandia zebra	14	20	chordates	animals
Amphilophus citrinellus	13	14	chordates	animals
Neolamprologus brichardi	14	21	chordates	animals
Astatotilapia calliptera	13	26	chordates	animals
Haplochromis burtoni	14	28	chordates	animals
Oreochromis niloticus	13	71	chordates	animals
Sander lucioperca	13	63	chordates	animals
Gasterosteus aculeatus	12	17	chordates	animals
Cottoperca gobio	10	57	chordates	animals
Cyclopterus lumpus	13	57	chordates	animals
Oryzias sinensis	12	41	chordates	animals
Oryzias latipes	14	33	chordates	animals
Nothobranchius furzeri	11	49	chordates	animals
Kryptolebias marmoratus	12	20	chordates	animals
Cyprinodon variegatus	14	28	chordates	animals
Gambusia affinis	14	27	chordates	animals
Xiphophorus maculatus	14	32	chordates	animals
Poecilia reticulata	13	23	chordates	animals
Fundulus heteroclitus	14	38	chordates	animals
Gadus morhua	13	14	chordates	animals
Hucho hucho	21	73	chordates	animals
Salmo salar	20	126	chordates	animals
Oncorhynchus kisutch	21	117	chordates	animals
Esox lucius	13	48	chordates	animals
Electrophorus electricus	12	52	chordates	animals
Ictalurus punctatus	11	22	chordates	animals
Pygocentrus nattereri	13	24	chordates	animals
Astyanax mexicanus	12	19	chordates	animals
Sinocyclocheilus grahami	21	62	chordates	animals
Cyprinus carpio	22	105	chordates	animals
Carassius auratus	24	132	chordates	animals
Danio rerio	13	25	chordates	animals
Denticeps clupeoides	12	82	chordates	animals
Clupea harengus	9	43	chordates	animals
Lepisosteus oculatus	11	14	chordates	animals
Latimeria chalumnae	10	13	chordates	animals
Callorhinchus milii	9	62	chordates	animals
Eptatretus burgeri	9	30	chordates	animals
Petromyzon marinus	8	8	chordates	animals
Ciona intestinalis	5	6	chordates	animals

Ephemera danica	7	7	insects	animals
Cloeon dipterum	5	11	insects	animals
Chrysoperla carnea	5	15	insects	animals
Megalurothrips usitatus	6	6	insects	animals
Thrips palmi	7	20	insects	animals
Frankliniella occidentalis	7	26	insects	animals
Ischnura elegans	6	12	insects	animals
Ladona fulva	6	6	insects	animals
Pediculus humanus	5	5	insects	animals
Timema podura	7	7	insects	animals
Zootermopsis nevadensis	7	14	insects	animals
Cryptotermes secundus	6	18	insects	animals
Coptotermes formosanus	6	6	insects	animals
Ctenocephalides felis	6	8	insects	animals
Colletes gigas	4	18	insects	animals
Neodiprion lecontei	4	23	insects	animals
Orussus abietinus	4	16	insects	animals
Cephus cinctus	4	21	insects	animals
Osmia lignaria	4	24	insects	animals
Megachile rotundata	4	10	insects	animals
Ceratosolen solmsi	4	5	insects	animals
Nomia melanderi	4	22	insects	animals
Dufourea novaeangliae	4	4	insects	animals
Megalopta genalis	4	27	insects	animals
Leptopilina heterotoma	5	21	insects	animals
Athalia rosae	4	22	insects	animals
Aphidius gifuensis	4	16	insects	animals
Chelonus insularis	4	20	insects	animals
Diachasma alloeum	4	4	insects	animals
Fopius arisanus	4	15	insects	animals
Cotesia congregata	2	2	insects	animals
Venturia canescens	4	21	insects	animals
Copidosoma floridanum	4	14	insects	animals
Ooceraea biroi	5	18	insects	animals
Cataglyphis hispanica	5	17	insects	animals
Pseudoatta argentina	5	5	insects	animals
Nylanderia fulva	4	5	insects	animals
Harpegnathos saltator	4	14	insects	animals
Dinoponera quadriceps	5	17	insects	animals
Odontomachus brunneus	4	24	insects	animals
Cyphomyrmex costatus	5	5	insects	animals
Vollenhovia emeryi	5	17	insects	animals
Monomorium pharaonis	5	13	insects	animals
Temnothorax longispinosus	5	5	insects	animals
Pseudomyrmex gracilis	4	16	insects	animals
Pogonomyrmex barbatus	5	6	insects	animals

Camponotus floridanus	5	7	insects	animals
Acromyrmex echinatior	5	16	insects	animals
Linepithema humile	5	23	insects	animals
Formica exsecta	5	19	insects	animals
Lasius niger	5	5	insects	animals
Wasmannia auropunctata	5	21	insects	animals
Trachymyrmex septentrionalis	5	16	insects	animals
Solenopsis invicta	5	15	insects	animals
Atta cephalotes	5	5	insects	animals
Trichogramma pretiosum	4	16	insects	animals
Habropoda laboriosa	3	3	insects	animals
Frieseomelitta varia	4	24	insects	animals
Eufriesea mexicana	4	18	insects	animals
Melipona quadrifasciata	3	3	insects	animals
Ceratina calcarata	4	17	insects	animals
Bombus terrestris	4	17	insects	animals
Apis mellifera	4	19	insects	animals
Polistes dominula	4	9	insects	animals
Polistes fuscatus	4	9	insects	animals
Vespula vulgaris	4	19	insects	animals
Vespa mandarinia	4	17	insects	animals
Nasonia vitripennis	4	34	insects	animals
Hermetia illucens	6	15	insects	animals
Clunio marinus	5	7	insects	animals
Polypedilum vanderplanki	5	7	insects	animals
Chironomus riparius	6	8	insects	animals
Contarinia nasturtii	5	12	insects	animals
Teleopsis dalmanni	10	12	insects	animals
Bradysia coprophila	7	21	insects	animals
Glossina fuscipes	6	19	insects	animals
Lucilia cuprina	6	10	insects	animals
Stomoxys calcitrans	6	19	insects	animals
Musca domestica	6	12	insects	animals
Scaptodrosophila lebanonensis	5	10	insects	animals
Drosophila suzukii	5	11	insects	animals
Drosophila melanogaster	5	19	insects	animals
Rhagoletis zephyria	9	22	insects	animals
Rhagoletis pomonella	7	14	insects	animals
Zeugodacus cucurbitae	6	25	insects	animals
Bactrocera oleae	6	25	insects	animals
Bactrocera tryoni	6	16	insects	animals
Bactrocera dorsalis	6	28	insects	animals
Ceratitis capitata	6	20	insects	animals
Culex pipiens	6	14	insects	animals
Anopheles sinensis	5	5	insects	animals
Anopheles gambiae	5	6	insects	animals

Aedes aegypti	6	17	insects	animals
Hyposmocoma kahamanoa	6	12	insects	animals
Arctia plantaginis	6	8	insects	animals
Eumeta japonica	8	8	insects	animals
Aricia agestis	6	15	insects	animals
Parnassius apollo	6	10	insects	animals
Papilio xuthus	5	9	insects	animals
Plutella xylostella	6	16	insects	animals
Chilo suppressalis	6	6	insects	animals
Diatraea saccharalis	6	10	insects	animals
Maniola jurtina	6	19	insects	animals
Nymphalis io	6	21	insects	animals
Pararge aegeria	6	8	insects	animals
Melitaea cinxia	6	6	insects	animals
Bicyclus anynana	5	22	insects	animals
Vanessa atalanta	6	14	insects	animals
Danaus plexippus	6	14	insects	animals
Amyelois transitella	6	12	insects	animals
Plodia interpunctella	6	21	insects	animals
Galleria mellonella	6	8	insects	animals
Manduca sexta	7	17	insects	animals
Leptidea sinapis	7	13	insects	animals
Colias croceus	6	14	insects	animals
Zerene cesonia	6	13	insects	animals
Pieris brassicae	6	17	insects	animals
Helicoverpa armigera	6	23	insects	animals
Helicoverpa zea	6	22	insects	animals
Trichoplusia ni	6	8	insects	animals
Spodoptera litura	6	9	insects	animals
Spodoptera frugiperda	6	22	insects	animals
Bombyx mori	6	20	insects	animals
Ignelater luminosus	5	5	insects	animals
Agrilus planipennis	6	9	insects	animals
Anoplophora glabripennis	6	11	insects	animals
Onthophagus taurus	6	7	insects	animals
Aethina tumida	6	9	insects	animals
Nicrophorus vespilloides	6	10	insects	animals
Harmonia axyridis	5	11	insects	animals
Coccinella septempunctata	5	11	insects	animals
Acanthoscelides obtectus	5	23	insects	animals
Callosobruchus maculatus	6	11	insects	animals
Gonioctena quinquepunctata	6	6	insects	animals
Diabrotica virgifera	5	7	insects	animals
Leptinotarsa decemlineata	5	7	insects	animals
Asbolus verrucosus	7	7	insects	animals
Tribolium castaneum	6	7	insects	animals

Tenebrio molitor	6	6	insects	animals
Abscondita terminalis	7	7	insects	animals
Lamprigera yunnana	7	7	insects	animals
Photinus pyralis	8	12	insects	animals
Dendroctonus ponderosae	6	24	insects	animals
Sitophilus oryzae	6	15	insects	animals
Halyomorpha halys	7	24	insects	animals
Nesidiocoris tenuis	7	7	insects	animals
Apolygus lucorum	7	7	insects	animals
Homalodisca vitripennis	8	26	insects	animals
Diaphorina citri	11	11	insects	animals
Nilaparvata lugens	7	11	insects	animals
Cimex lectularius	7	26	insects	animals
Daktulosphaira vitifoliae	6	18	insects	animals
Bemisia tabaci	8	10	insects	animals
Melanaphis sacchari	7	19	insects	animals
Cinara cedri	7	8	insects	animals
Sipha flava	7	13	insects	animals
Diuraphis noxia	6	13	insects	animals
Aphis gossypii	7	31	insects	animals
Rhopalosiphum maidis	16	63	insects	animals
Myzus persicae	7	25	insects	animals
Acyrthosiphon pisum	11	29	insects	animals
Gryllus bimaculatus	7	14	insects	animals
Ditylenchus destructor	8	8	nematodes	animals
Bursaphelenchus xylophilus	6	6	nematodes	animals
Caenorhabditis elegans	9	17	nematodes	animals
Stentor coeruleus	7	7	ciliates	ciliates
Stylonychia lemnae	9	9	ciliates	ciliates
Pseudocohnilembus persalinus	9	9	ciliates	ciliates
Tetrahymena thermophila SB210	7	7	ciliates	ciliates
Ichthyophthirius multifiliis	5	5	ciliates	ciliates
Paramecium tetraurelia	13	13	ciliates	ciliates
Theileria orientalis	3	3	apicomplexans	apicomplexans
Theileria parva	3	3	apicomplexans	apicomplexans
Babesia bigemina	3	3	apicomplexans	apicomplexans
Babesia bovis	2	2	apicomplexans	apicomplexans
Plasmodium falciparum CAMP/Malaysia	3	3	apicomplexans	apicomplexans
Gregarina niphandrodes	2	2	apicomplexans	apicomplexans
Cystoisospora suis	5	5	apicomplexans	apicomplexans
Toxoplasma gondii VEG	5	5	apicomplexans	apicomplexans
Hammondia hammondi	5	5	apicomplexans	apicomplexans
Besnoitia besnoiti	5	5	apicomplexans	apicomplexans
Cyclospora cayetanensis	4	4	apicomplexans	apicomplexans
Eimeria acervulina	3	3	apicomplexans	apicomplexans

Naegleria gruberi	5	5	other eukaryotes	other eukaryotes
Planoprotostelium fungivorum	11	11	other eukaryotes	other eukaryotes
Cavenderia fasciculata	3	3	other eukaryotes	other eukaryotes
Tieghemostelium lacteum	5	5	other eukaryotes	other eukaryotes
Dictyostelium purpureum	4	4	other eukaryotes	other eukaryotes
Entamoeba histolytica	1	1	other eukaryotes	other eukaryotes
Spironucleus salmonicida	1	1	diplomonads	diplomonads
Giardia intestinalis	1	1	diplomonads	diplomonads
Perkinsela sp. CCAP 1560/4	3	3	kinetoplastids	kinetoplastids
Phytomonas sp. EM1	4	4	kinetoplastids	kinetoplastids
Angomonas deanei	11	11	kinetoplastids	kinetoplastids
Strigomonas culicis	5	5	kinetoplastids	kinetoplastids
Trypanosoma equiperdum	4	4	kinetoplastids	kinetoplastids
Leptomonas seymouri	4	4	kinetoplastids	kinetoplastids
Leishmania donovani	4	4	kinetoplastids	kinetoplastids
Paramicrosporidium saccamoebae	3	3	other fungi	fungi
Rozella allomycis CSF55	4	4	other fungi	fungi
Paraphysoderma sedebokerense	6	6	other fungi	fungi
Catenaria anguillulae PL171	4	4	blastocladiomycetes	fungi
Allomyces macrogynus ATCC 38327	9	11	blastocladiomycetes	fungi
Amphiamblys sp. WSBS2006	3	3	microsporidians	fungi
Mitosporidium daphniae	3	3	microsporidians	fungi
Spraguea lophii 42_110	2	2	microsporidians	fungi
Edhazardia aedis USNM 41457	2	2	microsporidians	fungi
Nematocida parisii ERTm1	2	2	microsporidians	fungi
Enterospora canceri	2	2	microsporidians	fungi
Hepatospora eriocheir	2	2	microsporidians	fungi
Enterocytozoon bieneusi H348	2	2	microsporidians	fungi
Anncaliia algerae PRA109	3	3	microsporidians	fungi
Tubulinosema ratisbonensis	2	2	microsporidians	fungi
Encephalitozoon hellem ATCC 50504	2	2	microsporidians	fungi
Ordospora colligata	2	2	microsporidians	fungi
Hamiltosporidium magnivora	2	2	microsporidians	fungi
Pseudoloma neurophilia	3	3	microsporidians	fungi
Vavraia culicis subsp. floridensis	2	2	microsporidians	fungi
Trachipleistophora hominis	2	2	microsporidians	fungi
Nosema apis BRL 01	3	3	microsporidians	fungi
Vittaforma corneae ATCC 50505	2	2	microsporidians	fungi
Nosema ceranae	2	2	microsporidians	fungi
Moniliella sp. MCA 3643	8	8	basidiomycete fungi	fungi
Mixia osmundae IAM 14324	6	6	basidiomycete fungi	fungi
Dacrymyces fennicus	6	6	basidiomycete fungi	fungi
Dacryopinax primogenitus	5	5	basidiomycete fungi	fungi
Calocera viscosa TUFC12733	6	6	basidiomycete fungi	fungi
Unilacryma unispora	8	8	basidiomycete fungi	fungi

Cerinomyces crustulinus	6	6	basidiomycete fungi	fungi
Wallemia ichthyophaga	5	5	basidiomycete fungi	fungi
Malassezia restricta	5	5	basidiomycete fungi	fungi
Chionosphaera apobasidialis	6	6	basidiomycete fungi	fungi
Violaceomyces palustris	7	7	basidiomycete fungi	fungi
Urocystis occulta	6	6	basidiomycete fungi	fungi
Melanotaenium endogenum	6	6	basidiomycete fungi	fungi
Testicularia cyperi	6	6	basidiomycete fungi	fungi
Ustanciosporium gigantosporum	6	6	basidiomycete fungi	fungi
Kalmanozyma brasiliensis GHG001	6	6	basidiomycete fungi	fungi
Ustilago trichophora	10	10	basidiomycete fungi	fungi
Moesziomyces aphidis	5	5	basidiomycete fungi	fungi
Pseudozyma flocculosa	6	6	basidiomycete fungi	fungi
Sporisorium scitamineum	6	6	basidiomycete fungi	fungi
Septobasidium sp. PNB30-8B	7	7	basidiomycete fungi	fungi
Melampsora allii-populina	6	6	basidiomycete fungi	fungi
Puccinia sorghi	6	6	basidiomycete fungi	fungi
Pseudomicrostroma glucosiphilum	6	6	basidiomycete fungi	fungi
Jaminaea rosea	6	6	basidiomycete fungi	fungi
Tilletiaria anomala UBC 951	6	6	basidiomycete fungi	fungi
Ceraceosorus bombacis	6	6	basidiomycete fungi	fungi
Exobasidium maculosum	7	7	basidiomycete fungi	fungi
Meira miltonrushii	5	5	basidiomycete fungi	fungi
Acaromyces ingoldii	5	5	basidiomycete fungi	fungi
Heterodoassansia hygrophilae	5	5	basidiomycete fungi	fungi
Rhamphospora nymphaeae	5	5	basidiomycete fungi	fungi
Tilletiopsis washingtonensis	6	6	basidiomycete fungi	fungi
Robbauera albescens	6	6	basidiomycete fungi	fungi
Tilletia indica	6	6	basidiomycete fungi	fungi
Tilletia controversa	2	2	basidiomycete fungi	fungi
Naohidea sebacea CBS 8477	6	6	basidiomycete fungi	fungi
Erythrobasidium hasegawianum ATCC 9536	6	6	basidiomycete fungi	fungi
Symmetrospora gracilis	6	6	basidiomycete fungi	fungi
Intextomyces contiguus	9	9	basidiomycete fungi	fungi
Sclerogaster hysterangioides	8	8	basidiomycete fungi	fungi
Hysterangium stoloniferum HS.BST	8	8	basidiomycete fungi	fungi
Exidia glandulosa HHB12029	11	11	basidiomycete fungi	fungi
Elmerina caryae	8	8	basidiomycete fungi	fungi
Oliveonia pauxilla	8	8	basidiomycete fungi	fungi
Serendipita vermifera MAFF 305830	6	6	basidiomycete fungi	fungi
Jaapia argillacea MUCL 33604	7	7	basidiomycete fungi	fungi
Thelephora ganbajun	8	8	basidiomycete fungi	fungi
Xenasmatella vaga	8	8	basidiomycete fungi	fungi
Mutinus elegans	7	7	basidiomycete fungi	fungi
Plicaturopsis crispa FD-325 SS-3	1	1	basidiomycete fungi	fungi

Anomoloma albolutescens	5	5	basidiomycete fungi	fungi
Serpulomyces borealis	6	6	basidiomycete fungi	fungi
Anomoporia bombycina	6	6	basidiomycete fungi	fungi
Amylocorticium subincarnatum	6	6	basidiomycete fungi	fungi
Sistotremastrum suecicum HHB10207 ss-3	7	7	basidiomycete fungi	fungi
Porpomyces mucidus	6	6	basidiomycete fungi	fungi
Digitatispora marina	9	9	basidiomycete fungi	fungi
Fibularhizoctonia sp. CBS 109695	2	2	basidiomycete fungi	fungi
Piloderma byssinum	9	9	basidiomycete fungi	fungi
Phlebiella tulasnelloidea	8	8	basidiomycete fungi	fungi
Kurtia argillacea	6	6	basidiomycete fungi	fungi
Crustoderma dryinum	8	8	basidiomycete fungi	fungi
Mycobernardia incrustans	6	6	basidiomycete fungi	fungi
Sistotrema brinkmannii	6	6	basidiomycete fungi	fungi
Gautieria morchelliformis	6	6	basidiomycete fungi	fungi
Cantharellales sp. MUCL 035158	6	6	basidiomycete fungi	fungi
Hydnum rufescens UP504	6	6	basidiomycete fungi	fungi
Tulasnella calospora MUT 4182	8	9	basidiomycete fungi	fungi
Botryobasidium botryosum FD-172 SS1	6	6	basidiomycete fungi	fungi
Ceratobasidium theobromae	7	7	basidiomycete fungi	fungi
Rhizoctonia solani	7	7	basidiomycete fungi	fungi
Thanatephorus cucumeris	6	6	basidiomycete fungi	fungi
Sphaerobolus stellatus SS14	7	7	basidiomycete fungi	fungi
Geastrum triplex	8	8	basidiomycete fungi	fungi
Scytinostroma sp.	7	7	basidiomycete fungi	fungi
Vararia minispora EC-137	7	7	basidiomycete fungi	fungi
Peniophora sp. CONT	7	7	basidiomycete fungi	fungi
Bondarzewia mesenterica	7	7	basidiomycete fungi	fungi
Heterobasidion irregulare TC 32-1	7	7	basidiomycete fungi	fungi
Gloeopeniophorella convolvens	7	7	basidiomycete fungi	fungi
Dentipellis fragilis	7	7	basidiomycete fungi	fungi
Hericium coralloides	7	7	basidiomycete fungi	fungi
Laxitextum bicolor	7	7	basidiomycete fungi	fungi
Stereum hirsutum FP-91666 SS1	1	1	basidiomycete fungi	fungi
Boreostereum radiatum	7	7	basidiomycete fungi	fungi
Amylostereum chailletii	7	7	basidiomycete fungi	fungi
Lactifluus	7	7	basidiomycete fungi	fungi
Multifurca ochricompacta	6	6	basidiomycete fungi	fungi
Lactarius deliciosus	7	7	basidiomycete fungi	fungi
Russula compacta	8	8	basidiomycete fungi	fungi
Echinodontium tinctorium	7	7	basidiomycete fungi	fungi
Lentinellus vulpinus	7	7	basidiomycete fungi	fungi
Auriscalpium vulgare	8	8	basidiomycete fungi	fungi
Melanogaster broomeanus MBLB.BST	8	8	basidiomycete fungi	fungi

Pisolithus microcarpus 441	7	7	basidiomycete fungi	fungi
Hydnomerulius pinastri MD-312	4	4	basidiomycete fungi	fungi
Paxillus ammoniavirescens	6	6	basidiomycete fungi	fungi
Paxillus involutus ATCC 200175	5	5	basidiomycete fungi	fungi
Serpula lacrymans var. lacrymans S7.9	8	8	basidiomycete fungi	fungi
Coniophora olivacea	6	6	basidiomycete fungi	fungi
Hygrophoropsis aurantiaca	6	6	basidiomycete fungi	fungi
Scleroderma citrinum	6	6	basidiomycete fungi	fungi
Suillus subaureus	6	6	basidiomycete fungi	fungi
Rhizopogon vulgaris	7	7	basidiomycete fungi	fungi
Chalciporus piperatus	6	6	basidiomycete fungi	fungi
Imleria badia	6	6	basidiomycete fungi	fungi
Boletus edulis	8	8	basidiomycete fungi	fungi
Resinicium bicolor	6	6	basidiomycete fungi	fungi
Sidera vulgaris	7	7	basidiomycete fungi	fungi
Rickenella mellea	8	8	basidiomycete fungi	fungi
Pyrrhoderma noxium	7	7	basidiomycete fungi	fungi
Porodaedalea niemelaei	7	7	basidiomycete fungi	fungi
Phellinidium ferrugineofuscum	8	8	basidiomycete fungi	fungi
Fuscoporia viticola	5	5	basidiomycete fungi	fungi
Phellopilus nigrolimitatus	7	7	basidiomycete fungi	fungi
Phellinus igniarius	8	8	basidiomycete fungi	fungi
Schizopora paradoxa	7	7	basidiomycete fungi	fungi
Skeletocutis biguttulata	8	8	basidiomycete fungi	fungi
Laetiporus sulphureus 93-53	6	6	basidiomycete fungi	fungi
Obba rivulosa	7	7	basidiomycete fungi	fungi
Gelatoporia subvermispora B	7	7	basidiomycete fungi	fungi
Wolfiporia cocos MD-104 SS10	6	6	basidiomycete fungi	fungi
Fibroporia radiculosa	6	6	basidiomycete fungi	fungi
Epithele typhae	10	10	basidiomycete fungi	fungi
Climacocystis borealis	6	6	basidiomycete fungi	fungi
Meripilus sumstinei	6	6	basidiomycete fungi	fungi
Rigidoporus microporus	7	7	basidiomycete fungi	fungi
Pycnoporellus fulgens	6	6	basidiomycete fungi	fungi
Postia stiptica	8	8	basidiomycete fungi	fungi
Amylocystis lapponica	7	7	basidiomycete fungi	fungi
Sparassis crispa	6	6	basidiomycete fungi	fungi
Abortiporus biennis	6	6	basidiomycete fungi	fungi
Phlebiopsis gigantea 11061_1 CR5-6	9	9	basidiomycete fungi	fungi
Phanerochaete chrysosporium RP-78	9	9	basidiomycete fungi	fungi
Porostereum spadiceum	10	10	basidiomycete fungi	fungi
Hermanssonia centrifuga	6	6	basidiomycete fungi	fungi
Cabalodontia subcretacea	8	8	basidiomycete fungi	fungi
Antrodiella citrinella	5	5	basidiomycete fungi	fungi

Steccherinum ochraceum	7	7	basidiomycete fungi	fungi
Xanthoporus peckianus	6	6	basidiomycete fungi	fungi
Cytidiella melzeri	12	12	basidiomycete fungi	fungi
Flavodon flavus	7	7	basidiomycete fungi	fungi
Irpex rosettiformis	7	7	basidiomycete fungi	fungi
Leptoporus mollis	7	7	basidiomycete fungi	fungi
Ceriporia viridans	7	7	basidiomycete fungi	fungi
Daedalea quercina L-15889	6	6	basidiomycete fungi	fungi
Fomitopsis schrenkii	6	6	basidiomycete fungi	fungi
Neoantrodia serialis	8	8	basidiomycete fungi	fungi
Rhodofomes roseus	6	6	basidiomycete fungi	fungi
Poriella subacida	8	8	basidiomycete fungi	fungi
Cerioporus squamosus	9	9	basidiomycete fungi	fungi
Lentinus tigrinus ALCF2SS1-7	9	9	basidiomycete fungi	fungi
Ganoderma sinense ZZ0214-1	12	12	basidiomycete fungi	fungi
Polyporus brumalis	8	8	basidiomycete fungi	fungi
Dichomitus squalens	8	8	basidiomycete fungi	fungi
Trametes cinnabarina	6	6	basidiomycete fungi	fungi
Grifola frondosa	7	7	basidiomycete fungi	fungi
Neolentinus lepideus HHB14362 ss-1	7	7	basidiomycete fungi	fungi
Veluticeps abietina	6	6	basidiomycete fungi	fungi
Gloeophyllum trabeum ATCC 11539	8	8	basidiomycete fungi	fungi
Heliocybe sulcata	7	7	basidiomycete fungi	fungi
Clitocybe sp. CONT 1119283	6	6	basidiomycete fungi	fungi
Cristinia sonorae	6	6	basidiomycete fungi	fungi
Typhula sp. TRa3160C	7	7	basidiomycete fungi	fungi
Pterula gracilis	6	6	basidiomycete fungi	fungi
Pleurotus ostreatus PC15	6	6	basidiomycete fungi	fungi
Fistulina hepatica ATCC 64428	9	9	basidiomycete fungi	fungi
Dendrothele microspora	8	8	basidiomycete fungi	fungi
Laccaria bicolor S238N-H82	6	6	basidiomycete fungi	fungi
Cystostereum murrayi	8	8	basidiomycete fungi	fungi
Hygrocybe coccinea	7	7	basidiomycete fungi	fungi
Cortinarius saniosus	10	10	basidiomycete fungi	fungi
Phaeosolenia platensis	6	6	basidiomycete fungi	fungi
Aphanobasidium pseudotsugae	7	7	basidiomycete fungi	fungi
Radulomyces confluens	7	7	basidiomycete fungi	fungi
Hebeloma cylindrosporum h7	7	7	basidiomycete fungi	fungi
Gymnopilus dilepis	7	7	basidiomycete fungi	fungi
Moniliophthora roreri	6	6	basidiomycete fungi	fungi
Panaeolus cyanescens	6	6	basidiomycete fungi	fungi
Pluteus cervinus	6	6	basidiomycete fungi	fungi
Nia vibrissa	6	6	basidiomycete fungi	fungi
Flagelloscypha sp. PMI_526	6	6	basidiomycete fungi	fungi
Tricholoma populinum	5	5	basidiomycete fungi	fungi

Crepidotus cesatii	7	7	basidiomycete fungi	fungi
Schizophyllum commune H4-8	7	7	basidiomycete fungi	fungi
Auriculariopsis ampla	7	7	basidiomycete fungi	fungi
Lycoperdon perlatum	10	10	basidiomycete fungi	fungi
Amanita rubescens	7	7	basidiomycete fungi	fungi
Candolleomyces aberdarensis	4	4	basidiomycete fungi	fungi
Coprinopsis marcescibilis	6	6	basidiomycete fungi	fungi
Coprinellus micaceus	8	8	basidiomycete fungi	fungi
Pholiota sp.	8	8	basidiomycete fungi	fungi
Hypholoma sublateritium FD-334 SS-4	8	8	basidiomycete fungi	fungi
Galerina marginata CBS 339.88	7	7	basidiomycete fungi	fungi
Psilocybe cyanescens	8	8	basidiomycete fungi	fungi
Agrocybe praecox	7	7	basidiomycete fungi	fungi
Mycocalia denudata	6	6	basidiomycete fungi	fungi
Cyathus stercoreus	8	8	basidiomycete fungi	fungi
Crucibulum laeve	7	7	basidiomycete fungi	fungi
Guyanagaster necrorhizus MCA 3950	10	10	basidiomycete fungi	fungi
Cylindrobasidium torrendii FP15055 ss-10	9	9	basidiomycete fungi	fungi
Desarmillaria ectypa	9	9	basidiomycete fungi	fungi
Armillaria borealis	11	11	basidiomycete fungi	fungi
Termitomyces sp. J132	6	6	basidiomycete fungi	fungi
Hypsizygus marmoreus	8	8	basidiomycete fungi	fungi
Favolaschia claudopus	18	18	basidiomycete fungi	fungi
Roridomyces roridus	8	8	basidiomycete fungi	fungi
Mycena galericulata	11	11	basidiomycete fungi	fungi
Panellus stipticus	7	7	basidiomycete fungi	fungi
Conocybe apala	7	7	basidiomycete fungi	fungi
Bolbitius vitellinus	7	7	basidiomycete fungi	fungi
Cyclocybe aegerita	6	6	basidiomycete fungi	fungi
Gymnopus androsaceus JB14	7	7	basidiomycete fungi	fungi
Collybiopsis luxurians FD-317 M1	2	2	basidiomycete fungi	fungi
Rhodocollybia butyracea	5	5	basidiomycete fungi	fungi
Lentinula edodes	5	5	basidiomycete fungi	fungi
Agaricus bisporus	6	6	basidiomycete fungi	fungi
Phleogena faginea	9	9	basidiomycete fungi	fungi
Meredithblackwellia eburnea MCA 4105	8	8	basidiomycete fungi	fungi
Microbotryum intermedium	6	6	basidiomycete fungi	fungi
Hyalopycnis blepharistoma	6	6	basidiomycete fungi	fungi
Slooffia pilatii	9	9	basidiomycete fungi	fungi
Leucosporidium creatinivorum	7	7	basidiomycete fungi	fungi
Rhodotorula toruloides	8	8	basidiomycete fungi	fungi
Sporobolomyces pararoseus	7	7	basidiomycete fungi	fungi
Mrakia frigida	8	8	basidiomycete fungi	fungi
Phaffia rhodozyma	6	6	basidiomycete fungi	fungi

Pascua guehoae	7	7	basidiomycete fungi	fungi
Trichosporon asahii var. asahii CBS 2479	5	5	basidiomycete fungi	fungi
Cutaneotrichosporon oleaginosum	6	6	basidiomycete fungi	fungi
Haglerozyma chiarellii	6	6	basidiomycete fungi	fungi
Apiotrichum porosum	5	5	basidiomycete fungi	fungi
Vanrija humicola	4	4	basidiomycete fungi	fungi
Solicoccozyma terrea	7	7	basidiomycete fungi	fungi
Filobasidium floriforme	6	6	basidiomycete fungi	fungi
Saitozyma podzolica	6	6	basidiomycete fungi	fungi
Kwoniella pini CBS 10737	6	6	basidiomycete fungi	fungi
Cryptococcus neoformans var. grubii	7	7	basidiomycete fungi	fungi
Naematelia encephala	5	5	basidiomycete fungi	fungi
Sirobasidium intermedium	6	6	basidiomycete fungi	fungi
Fibulobasidium inconspicuum	6	6	basidiomycete fungi	fungi
Papiliotrema laurentii	6	6	basidiomycete fungi	fungi
Tremella mesenterica	5	5	basidiomycete fungi	fungi
Sterigmatosporidium polymorphum	7	7	basidiomycete fungi	fungi
Fellomyces penicillatus	6	6	basidiomycete fungi	fungi
Kockovaella imperatae	6	6	basidiomycete fungi	fungi
Sclerophora sanguinea	4	4	ascomycete fungi	fungi
Neolecta irregularis DAH-3	6	6	ascomycete fungi	fungi
Xylona heveae TC161	4	4	ascomycete fungi	fungi
Trinosporium guianense	4	4	ascomycete fungi	fungi
Pneumocystis murina B123	6	6	ascomycete fungi	fungi
Saitoella complicata NRRL Y-17804	6	6	ascomycete fungi	fungi
Schizosaccharomyces pombe 972h-	3	3	ascomycete fungi	fungi
Lasallia pustulata	3	3	ascomycete fungi	fungi
Usnea florida	4	4	ascomycete fungi	fungi
Dactylellina haptotyla CBS 200.50	4	4	ascomycete fungi	fungi
Orbilia oligospora	4	5	ascomycete fungi	fungi
Arthrobotrys flagrans	4	4	ascomycete fungi	fungi
Protomyces lactucae-debilis	5	5	ascomycete fungi	fungi
Plectania melastoma	5	5	ascomycete fungi	fungi
Ascodesmis nigricans	5	5	ascomycete fungi	fungi
Tirmania nivea	4	4	ascomycete fungi	fungi
Terfezia claveryi	4	4	ascomycete fungi	fungi
Kalaharituber pfeilii	4	4	ascomycete fungi	fungi
Mattirolomyces terfezioides	4	4	ascomycete fungi	fungi
Sarcoscypha coccinea	6	6	ascomycete fungi	fungi
Sphaerosporella brunnea	5	5	ascomycete fungi	fungi
Trichophaea hybrida	5	5	ascomycete fungi	fungi
Caloscypha fulgens	3	3	ascomycete fungi	fungi
Rhizina undulata	12	12	ascomycete fungi	fungi
Leucangium carthusianum	4	4	ascomycete fungi	fungi
Choiromyces venosus 120613-1	4	4	ascomycete fungi	fungi

Tuber magnatum	4	4	ascomycete fungi	fungi
Kalapuya brunnea	4	4	ascomycete fungi	fungi
Verpa conica	4	4	ascomycete fungi	fungi
Disciotis venosa	4	4	ascomycete fungi	fungi
Morchella esculenta	4	4	ascomycete fungi	fungi
Gyromitra esculenta	4	4	ascomycete fungi	fungi
Xylogone sp. PMI_703	5	5	ascomycete fungi	fungi
Leotiomycetes sp. MPI-SDFR-AT-0126	4	4	ascomycete fungi	fungi
Oidiodendron maius Zn	5	5	ascomycete fungi	fungi
Pseudogymnoascus verrucosus	5	5	ascomycete fungi	fungi
Antarctomyces sp.	4	4	ascomycete fungi	fungi
Thelebolus microsporus	4	4	ascomycete fungi	fungi
Coccomyces strobi	5	5	ascomycete fungi	fungi
Lophodermium piceae	6	6	ascomycete fungi	fungi
Bulgaria inquinans	4	4	ascomycete fungi	fungi
Golovinomyces cichoracearum	4	4	ascomycete fungi	fungi
Blumeria hordei	4	4	ascomycete fungi	fungi
Erysiphe necator	4	4	ascomycete fungi	fungi
Scytalidium lignicola	8	8	ascomycete fungi	fungi
Helotiales sp. DMI_Dod_QoI	4	4	ascomycete fungi	fungi
Venustampulla echinocandica	4	4	ascomycete fungi	fungi
Polyphilus sieberi	4	4	ascomycete fungi	fungi
Rhexocercosporidium sp. MPI-PUGE-AT-0058	4	4	ascomycete fungi	fungi
Calycina marina	5	5	ascomycete fungi	fungi
Helotiales sp.	6	6	ascomycete fungi	fungi
Rutstroemia firma	4	4	ascomycete fungi	fungi
Cadophora sp.	4	4	ascomycete fungi	fungi
Leptodontidium sp. 2 PMI_412	4	4	ascomycete fungi	fungi
Bisporella sp.	5	5	ascomycete fungi	fungi
Hymenoscyphus varicosporioides	4	4	ascomycete fungi	fungi
Glarea lozoyensis ATCC 20868	4	4	ascomycete fungi	fungi
Hyaloscypha bicolor E	5	5	ascomycete fungi	fungi
Diplocarpon rosae	6	6	ascomycete fungi	fungi
Drepanopeziza brunnea f. sp. 'multigermtubi' MB_m1	4	4	ascomycete fungi	fungi
Amorphotheca resinae ATCC 22711	5	5	ascomycete fungi	fungi
Coleophoma crateriformis	5	5	ascomycete fungi	fungi
Marssonina coronariae	4	4	ascomycete fungi	fungi
Halenospora varia	4	4	ascomycete fungi	fungi
Lachnellula willkommii	3	3	ascomycete fungi	fungi
Acephala macrosclerotiorum	4	4	ascomycete fungi	fungi
Phialocephala subalpina	4	4	ascomycete fungi	fungi
Mollisia scopiformis	4	4	ascomycete fungi	fungi
Loramyces juncicola	4	4	ascomycete fungi	fungi
Chalara hyalina	4	4	ascomycete fungi	fungi

Amylocarpus encephaloides	4	4	ascomycete fungi	fungi
Rhynchosporium secalis	4	4	ascomycete fungi	fungi
Botrytis cinerea B05.10	4	4	ascomycete fungi	fungi
Monilinia fructicola	3	4	ascomycete fungi	fungi
Sclerotinia sclerotiorum	4	4	ascomycete fungi	fungi
Melanospora tiffanyae	4	4	ascomycete fungi	fungi
Boliniaceae sp. AZ0804	4	4	ascomycete fungi	fungi
Chaetosphaeriaceae sp.	4	4	ascomycete fungi	fungi
Thozetella sp. PMI_491	5	5	ascomycete fungi	fungi
Acidothrix acidophila	4	4	ascomycete fungi	fungi
Phaeoacremonium minimum UCRPA7	4	4	ascomycete fungi	fungi
Thyridium curvatum	4	4	ascomycete fungi	fungi
Ophiostoma piceae UAMH 11346	4	4	ascomycete fungi	fungi
Sporothrix insectorum RCEF 264	4	4	ascomycete fungi	fungi
Grosmannia clavigera kw1407	5	5	ascomycete fungi	fungi
Torpedospora radiata	5	5	ascomycete fungi	fungi
Magnaporthiopsis poae ATCC 64411	4	4	ascomycete fungi	fungi
Gaeumannomyces tritici R3-111a-1	4	4	ascomycete fungi	fungi
Pyricularia oryzae	4	4	ascomycete fungi	fungi
Coniochaeta pulveracea	4	4	ascomycete fungi	fungi
Coniella lustricola	4	4	ascomycete fungi	fungi
Gnomoniopsis castaneae	4	4	ascomycete fungi	fungi
Cryptodiaporthe sp. AZ0812	4	4	ascomycete fungi	fungi
Diaporthaceae sp. PMI_573	4	4	ascomycete fungi	fungi
Diaporthe helianthi	4	4	ascomycete fungi	fungi
Cytospora leucostoma	4	4	ascomycete fungi	fungi
Lollipopaia minuta	4	4	ascomycete fungi	fungi
Valsa mali	4	4	ascomycete fungi	fungi
Lindra thalassiae	4	4	ascomycete fungi	fungi
Bretziella fagacearum	5	5	ascomycete fungi	fungi
Ceratocystis platani	4	4	ascomycete fungi	fungi
Thielaviopsis punctulata	5	5	ascomycete fungi	fungi
Corollospora maritima	5	5	ascomycete fungi	fungi
Microascus trigonosporus CBS 218.31	5	5	ascomycete fungi	fungi
Scedosporium apiospermum	3	3	ascomycete fungi	fungi
Lomentospora prolificans	5	5	ascomycete fungi	fungi
Xylariales sp. AK1849	4	4	ascomycete fungi	fungi
Pseudomassariella vexata	5	5	ascomycete fungi	fungi
Apiospora esporlensis	4	4	ascomycete fungi	fungi
Nigrospora oryzae	4	4	ascomycete fungi	fungi
Daldinia sp. EC12	4	4	ascomycete fungi	fungi
Hypoxylon vinosopulvinatum	4	4	ascomycete fungi	fungi
Microdochium bolleyi	4	4	ascomycete fungi	fungi
Monosporascus cannonballus	4	4	ascomycete fungi	fungi

Neopestalotiopsis sp.	4	4	ascomycete fungi	fungi
Pestalotiopsis fici W106-1	4	4	ascomycete fungi	fungi
Truncatella angustata	4	4	ascomycete fungi	fungi
Eutypa lata UCREL1	3	3	ascomycete fungi	fungi
Diatrype disciformis	4	4	ascomycete fungi	fungi
Kretzschmaria zonata	4	4	ascomycete fungi	fungi
Rosellinia necatrix	3	3	ascomycete fungi	fungi
Xylaria hypoxylon	4	4	ascomycete fungi	fungi
Sodiomyces alkalinus F11	4	4	ascomycete fungi	fungi
Plectosphaerella cucumerina	4	4	ascomycete fungi	fungi
Verticillium dahliae	4	4	ascomycete fungi	fungi
Colletotrichum trifolii	4	4	ascomycete fungi	fungi
Schizothecium vesticola	4	4	ascomycete fungi	fungi
Podospora appendiculata	4	4	ascomycete fungi	fungi
Madurella mycetomatis	4	4	ascomycete fungi	fungi
Lasiosphaeriaceae sp. AZ0830	4	4	ascomycete fungi	fungi
Apodospora peruviana	4	4	ascomycete fungi	fungi
Apiosordaria backusii	4	4	ascomycete fungi	fungi
Lasiosphaeris hirsuta	4	4	ascomycete fungi	fungi
Bombardia bombarda	4	4	ascomycete fungi	fungi
Lasiosphaeria ovina	3	3	ascomycete fungi	fungi
Cercophora newfieldiana	3	3	ascomycete fungi	fungi
Dichotomopilus funicola	4	4	ascomycete fungi	fungi
Thermothelomyces thermophilus ATCC 42464	4	4	ascomycete fungi	fungi
Chaetomium globosum	4	4	ascomycete fungi	fungi
Sordaria macrospora	4	4	ascomycete fungi	fungi
Neurospora crassa	4	4	ascomycete fungi	fungi
Emericellopsis atlantica	4	4	ascomycete fungi	fungi
Acremonium chrysogenum ATCC 11550	4	4	ascomycete fungi	fungi
Niesslia exilis	4	4	ascomycete fungi	fungi
Valetoniellopsis laxa	4	4	ascomycete fungi	fungi
Myrothecium inundatum	4	4	ascomycete fungi	fungi
Stachybotrys elegans	4	4	ascomycete fungi	fungi
Stanjemonium grisellum	4	4	ascomycete fungi	fungi
Akanthomyces lecanii	4	4	ascomycete fungi	fungi
Beauveria brongniartii RCEF 3172	4	4	ascomycete fungi	fungi
Beauveria bassiana	4	4	ascomycete fungi	fungi
Cordyceps javanica	4	4	ascomycete fungi	fungi
Hirsutella minnesotensis 3608	2	2	ascomycete fungi	fungi
Ophiocordyceps unilateralis	4	4	ascomycete fungi	fungi
Drechmeria coniospora	4	4	ascomycete fungi	fungi
Tolypocladium capitatum	4	4	ascomycete fungi	fungi
Purpureocillium lilacinum	4	4	ascomycete fungi	fungi
Nectriopsis sporangiicola NRRL 22127	4	4	ascomycete fungi	fungi

Clonostachys rosea	4	4	ascomycete fungi	fungi
Escovopsis weberi	4	4	ascomycete fungi	fungi
Trichoderma harzianum	4	4	ascomycete fungi	fungi
[Torrubiella] hemipterigena	4	4	ascomycete fungi	fungi
Ustilaginoidea virens	3	3	ascomycete fungi	fungi
Claviceps purpurea 20.1	4	4	ascomycete fungi	fungi
Moelleriella libera RCEF 2490	4	4	ascomycete fungi	fungi
Pochonia chlamydosporia 123	4	4	ascomycete fungi	fungi
Metarhizium anisopliae	4	4	ascomycete fungi	fungi
Mariannaea sp.	4	4	ascomycete fungi	fungi
Thelonectria olida	4	4	ascomycete fungi	fungi
Ilyonectria robusta	4	4	ascomycete fungi	fungi
Dactylonectria macrodidyma	4	4	ascomycete fungi	fungi
Neonectria ditissima	4	4	ascomycete fungi	fungi
Fusarium oxysporum	4	4	ascomycete fungi	fungi
Fusarium fujikuroi	4	4	ascomycete fungi	fungi
Endocarpon pusillum Z07020	4	4	ascomycete fungi	fungi
Phaeomoniella chlamydospora	4	4	ascomycete fungi	fungi
Chaetothyriales sp. CBS 134920	5	5	ascomycete fungi	fungi
Coccodinium bartschii CBS 121709	5	5	ascomycete fungi	fungi
Cyphellophora europaea CBS 101466	5	5	ascomycete fungi	fungi
Rhinocladiella mackenziei CBS 650.93	5	5	ascomycete fungi	fungi
Fonsecaea monophora	5	5	ascomycete fungi	fungi
Exophiala spinifera	5	5	ascomycete fungi	fungi
Capronia fungicola	5	5	ascomycete fungi	fungi
Phialophora americana	5	5	ascomycete fungi	fungi
Polytolypa hystricis UAMH7299	4	4	ascomycete fungi	fungi
Ascosphaera apis ARSEF 7405	4	4	ascomycete fungi	fungi
Paracoccidioides brasiliensis	4	4	ascomycete fungi	fungi
Helicocarpus griseus UAMH5409	4	4	ascomycete fungi	fungi
Emergomyces pasteurianus Ep9510	4	4	ascomycete fungi	fungi
Blastomyces dermatitidis ATCC 26199	4	4	ascomycete fungi	fungi
Histoplasma mississippiense (nom. inval.)	4	4	ascomycete fungi	fungi
Emmonsia crescens	4	4	ascomycete fungi	fungi
Microsporum canis CBS 113480	4	4	ascomycete fungi	fungi
Nannizzia gypsea CBS 118893	4	4	ascomycete fungi	fungi
Trichophyton rubrum	4	4	ascomycete fungi	fungi
Uncinocarpus reesii 1704	4	4	ascomycete fungi	fungi
Coccidioides immitis	4	4	ascomycete fungi	fungi
Elaphomyces granulatus	4	4	ascomycete fungi	fungi
Paecilomyces variotii	4	4	ascomycete fungi	fungi
Rasamsonia emersonii CBS 393.64	4	4	ascomycete fungi	fungi
Trichocoma paradoxa	4	4	ascomycete fungi	fungi
Talaromyces islandicus	4	4	ascomycete fungi	fungi

Penicilliopsis zonata CBS 506.65	4	4	ascomycete fungi	fungi
Monascus purpureus	4	4	ascomycete fungi	fungi
Penicillium brevicompactum	4	4	ascomycete fungi	fungi
Aspergillus flavus	5	5	ascomycete fungi	fungi
Gloniopsis sp. FL0384	4	4	ascomycete fungi	fungi
Valsariales	4	4	ascomycete fungi	fungi
Rachicladosporium antarcticum	10	10	ascomycete fungi	fungi
Lizonia empirigonia	4	4	ascomycete fungi	fungi
Eremomyces bilateralis CBS 781.70	5	5	ascomycete fungi	fungi
Rhizodiscina lignyota	4	4	ascomycete fungi	fungi
Aulographum hederae CBS 113979	4	4	ascomycete fungi	fungi
Coniosporium apollinis CBS 100218	5	5	ascomycete fungi	fungi
Viridothelium virens	4	4	ascomycete fungi	fungi
Stomiopeltis betulae	5	5	ascomycete fungi	fungi
Trichodelitschia bisporula	5	5	ascomycete fungi	fungi
Microthyrium microscopicum	5	5	ascomycete fungi	fungi
Lineolata rhizophorae	4	4	ascomycete fungi	fungi
Cenococcum geophilum 1.58	1	1	ascomycete fungi	fungi
Glonium stellatum	4	4	ascomycete fungi	fungi
Pseudovirgaria hyperparasitica	4	4	ascomycete fungi	fungi
Lepidopterella palustris CBS 459.81	4	4	ascomycete fungi	fungi
Mytilinidiaceae sp. NC0267	4	4	ascomycete fungi	fungi
Mytilinidion resinicola	4	4	ascomycete fungi	fungi
Lophium mytilinum	4	4	ascomycete fungi	fungi
Zopfia rhizophila CBS 207.26	4	4	ascomycete fungi	fungi
Didymocrea sadasivanii	4	4	ascomycete fungi	fungi
Cryomyces minteri	7	7	ascomycete fungi	fungi
Peltaster fructicola	4	4	ascomycete fungi	fungi
Halojulella avicenniae	4	4	ascomycete fungi	fungi
Jahnula aquatica	4	4	ascomycete fungi	fungi
Aliquandostipite khaoyaiensis	3	3	ascomycete fungi	fungi
Zasmidium cellare ATCC 36951	4	4	ascomycete fungi	fungi
Sphaerulina musiva SO2202	4	4	ascomycete fungi	fungi
Zymoseptoria tritici IPO323	4	4	ascomycete fungi	fungi
Cercospora beticola	5	5	ascomycete fungi	fungi
Pseudocercospora ulei	3	3	ascomycete fungi	fungi
Pseudocercospora musae	3	3	ascomycete fungi	fungi
Ramularia collo-cygni	4	4	ascomycete fungi	fungi
Teratosphaeriaceae sp.	4	4	ascomycete fungi	fungi
Baudoinia panamericana UAMH 10762	4	4	ascomycete fungi	fungi
Salinomyces thailandica	4	4	ascomycete fungi	fungi
Friedmanniomyces simplex	6	6	ascomycete fungi	fungi
Neohortaea acidophila	4	4	ascomycete fungi	fungi
Acidomyces sp. 'richmondensis'	4	4	ascomycete fungi	fungi
Teratosphaeria nubilosa	4	4	ascomycete fungi	fungi

Hortaea werneckii	8	8	ascomycete fungi	fungi
Myriangiaceae sp. NC1570	5	5	ascomycete fungi	fungi
Sphaceloma murrayae	4	4	ascomycete fungi	fungi
Elsinoe australis	4	4	ascomycete fungi	fungi
Aplosporella prunicola CBS 121167	5	5	ascomycete fungi	fungi
Melanops tulasnei	5	5	ascomycete fungi	fungi
Phyllosticta citricarpa	5	5	ascomycete fungi	fungi
Pseudofusicoccum sp. LGMF1611	5	5	ascomycete fungi	fungi
Microdiplodia sp. AK1800	4	4	ascomycete fungi	fungi
Neofusicoccum parvum UCRNP2	5	5	ascomycete fungi	fungi
Diplodia seriata	4	4	ascomycete fungi	fungi
Lasiodiplodia theobromae	5	5	ascomycete fungi	fungi
Macrophomina phaseolina	5	5	ascomycete fungi	fungi
Delphinella strobiligena	4	4	ascomycete fungi	fungi
Aureobasidium pullulans	4	4	ascomycete fungi	fungi
Teichospora sp. FL1471	4	4	ascomycete fungi	fungi
Flavomyces fulophazii	4	4	ascomycete fungi	fungi
Acrocalymma vagum	4	4	ascomycete fungi	fungi
Aquilomyces patris	4	4	ascomycete fungi	fungi
Aaosphaeria arxii CBS 175.79	4	4	ascomycete fungi	fungi
Corynespora cassiicola Philippines	4	4	ascomycete fungi	fungi
Paraphaeosphaeria sporulosa	4	4	ascomycete fungi	fungi
Bimuria novae-zelandiae CBS 107.79	4	4	ascomycete fungi	fungi
Amniculicola lignicola CBS 123094	4	4	ascomycete fungi	fungi
Dothidotthia symphoricarpi CBS 119687	4	4	ascomycete fungi	fungi
Herpotrichia sp. AK1299	4	4	ascomycete fungi	fungi
Melanomma pulvis-pyrius CBS 109.77	4	4	ascomycete fungi	fungi
Pleomassaria siparia CBS 279.74	4	4	ascomycete fungi	fungi
Lophiostoma macrostomum CBS 122681	4	4	ascomycete fungi	fungi
Darksidea alpha	4	4	ascomycete fungi	fungi
Lentithecium fluviatile CBS 122367	4	4	ascomycete fungi	fungi
Fenestella fenestrata	4	4	ascomycete fungi	fungi
Pyrenochaeta sp. DS3sAY3a	4	4	ascomycete fungi	fungi
Clathrospora elynae	4	4	ascomycete fungi	fungi
Lophiotrema nucula	4	4	ascomycete fungi	fungi
Polyplosphaeria fusca	4	4	ascomycete fungi	fungi
Clohesyomyces aquaticus	4	4	ascomycete fungi	fungi
Lindgomyces ingoldianus	4	4	ascomycete fungi	fungi
Neopyrenochaeta inflorescentiae	4	4	ascomycete fungi	fungi
Trematosphaeria pertusa	4	4	ascomycete fungi	fungi
Sporormia fimetaria CBS 119925	4	4	ascomycete fungi	fungi
Westerdykella ornata	4	4	ascomycete fungi	fungi
Pseudopyrenochaeta lycopersici	4	4	ascomycete fungi	fungi
Dendryphion nanum	4	4	ascomycete fungi	fungi

Phaeosphaeriaceae sp.	4	4	ascomycete fungi	fungi
Phaeosphaeria poagena	4	4	ascomycete fungi	fungi
Ophiobolus disseminans	4	4	ascomycete fungi	fungi
Paraphoma chrysanthemicola	4	4	ascomycete fungi	fungi
Parastagonospora nodorum SN15	4	4	ascomycete fungi	fungi
Setomelanomma holmii	4	4	ascomycete fungi	fungi
Massarina eburnea CBS 473.64	4	4	ascomycete fungi	fungi
Stagonospora sp. SRC1lsM3a	4	4	ascomycete fungi	fungi
Byssothecium circinans	4	4	ascomycete fungi	fungi
Massariosphaeria phaeospora	4	4	ascomycete fungi	fungi
Periconia macrospinosa	4	4	ascomycete fungi	fungi
Plenodomus lingam JN3	4	4	ascomycete fungi	fungi
Ampelomyces quisqualis	4	4	ascomycete fungi	fungi
Leptosphaeria microscopica	4	4	ascomycete fungi	fungi
Pleosporaceae sp. PMI_138	4	4	ascomycete fungi	fungi
Exserohilum turcica Et28A	4	4	ascomycete fungi	fungi
Bipolaris maydis ATCC 48331	4	4	ascomycete fungi	fungi
Decorospora gaudefroyi	4	4	ascomycete fungi	fungi
Stemphylium lycopersici	4	4	ascomycete fungi	fungi
Pyrenophora tritici-repentis	4	4	ascomycete fungi	fungi
Alternaria alternata	4	4	ascomycete fungi	fungi
Didymella exigua CBS 183.55	4	4	ascomycete fungi	fungi
Boeremia exigua	4	4	ascomycete fungi	fungi
Epicoccum nigrum	4	4	ascomycete fungi	fungi
Ectophoma multirostrata	4	4	ascomycete fungi	fungi
Ascochyta rabiei	4	4	ascomycete fungi	fungi
Tothia fuscella	5	5	ascomycete fungi	fungi
Verruconis gallopava	5	13	ascomycete fungi	fungi
Venturia inaequalis	3	3	ascomycete fungi	fungi
Kuraishia capsulata CBS 1993	5	5	ascomycete fungi	fungi
Ascoidea rubescens DSM 1968	5	5	ascomycete fungi	fungi
Babjeviella inositovora NRRL Y-12698	4	4	ascomycete fungi	fungi
Nadsonia fulvescens var. elongata DSM 6958	5	5	ascomycete fungi	fungi
Lipomyces starkeyi NRRL Y-11557	5	5	ascomycete fungi	fungi
Tortispora caseinolytica NRRL Y-17796	4	4	ascomycete fungi	fungi
Botryozyma mucatilis	5	5	ascomycete fungi	fungi
Myxozyma melibiosi	5	5	ascomycete fungi	fungi
Hanseniaspora uvarum	4	4	ascomycete fungi	fungi
[Candida] intermedia	4	4	ascomycete fungi	fungi
Metschnikowia bicuspidata	4	4	ascomycete fungi	fungi
Cephaloascus fragrans	5	5	ascomycete fungi	fungi
Sugiyamaella americana	5	5	ascomycete fungi	fungi
Blastobotrys attinorum	6	6	ascomycete fungi	fungi
Trichomonascus petasosporus	5	5	ascomycete fungi	fungi

Wickerhamiella sorbophila	4	4	ascomycete fungi	fungi
Zygoascus hellenicus	4	4	ascomycete fungi	fungi
Diutina rugosa	6	6	ascomycete fungi	fungi
Suhomyces tanzawaensis NRRL Y-17324	6	6	ascomycete fungi	fungi
Hyphopichia burtonii NRRL Y-1933	5	5	ascomycete fungi	fungi
Spathaspora passalidarum NRRL Y-27907	6	6	ascomycete fungi	fungi
Yamadazyma tenuis ATCC 10573	4	6	ascomycete fungi	fungi
Millerozyma farinosa CBS 7064	8	8	ascomycete fungi	fungi
Lodderomyces elongisporus NRRL YB-4239	6	6	ascomycete fungi	fungi
Scheffersomyces stipitis CBS 6054	6	6	ascomycete fungi	fungi
Meyerozyma guilliermondii ATCC 6260	5	5	ascomycete fungi	fungi
Debaryomyces fabryi	5	5	ascomycete fungi	fungi
Candida parapsilosis	6	6	ascomycete fungi	fungi
Geotrichum candidum	5	5	ascomycete fungi	fungi
Sporopachydermia lactativora	5	5	ascomycete fungi	fungi
Yarrowia lipolytica	5	5	ascomycete fungi	fungi
Kazachstania africana CBS 2517	5	5	ascomycete fungi	fungi
Tetrapisispora blattae CBS 6284	5	5	ascomycete fungi	fungi
Naumovozyma castellii CBS 4309	5	5	ascomycete fungi	fungi
Vanderwaltozyma polyspora DSM 70294	6	7	ascomycete fungi	fungi
Eremothecium cymbalariae DBVPG#7215	5	5	ascomycete fungi	fungi
[Ashbya] aceris (nom. inval.)	5	5	ascomycete fungi	fungi
Eremothecium gossypii ATCC 10895	5	5	ascomycete fungi	fungi
Zygotorulaspora mrakii	5	5	ascomycete fungi	fungi
Nakaseomyces glabratus	5	5	ascomycete fungi	fungi
Lachancea fermentati	5	5	ascomycete fungi	fungi
Zygosaccharomyces bailii	5	5	ascomycete fungi	fungi
Torulaspora globosa	5	5	ascomycete fungi	fungi
Torulaspora delbrueckii	5	5	ascomycete fungi	fungi
Saccharomyces cerevisiae	5	5	ascomycete fungi	fungi
Kluyveromyces marxianus	5	5	ascomycete fungi	fungi
[Candida] arabinofermentans NRRL YB-2248	4	4	ascomycete fungi	fungi
Ogataea polymorpha	4	4	ascomycete fungi	fungi
Brettanomyces bruxellensis	2	2	ascomycete fungi	fungi
[Candida] inconspicua	3	3	ascomycete fungi	fungi
Pichia kudriavzevii	3	3	ascomycete fungi	fungi
Wickerhamomyces anomalus NRRL Y-366-8	4	4	ascomycete fungi	fungi
Komagataella pastoris	3	3	ascomycete fungi	fungi
Cyberlindnera jadinii	1	1	ascomycete fungi	fungi
Basidiobolus meristosporus CBS 931.73	10	10	zoopagomycota	fungi
Ramicandelaber brevisporus	3	3	zoopagomycota	fungi

Dimargaris cristalligena	5	5	zoopagomycota	fungi
Syncephalis pseudoplumigaleata	3	3	zoopagomycota	fungi
Piptocephalis tieghemiana	3	3	zoopagomycota	fungi
Thamnocephalis sphaerospora	3	3	zoopagomycota	fungi
Neoconidiobolus thromboides FSU 785	6	6	zoopagomycota	fungi
Conidiobolus coronatus NRRL 28638	4	4	zoopagomycota	fungi
Entomophaga maimaiga	7	7	zoopagomycota	fungi
Zoophthora radicans	8	8	zoopagomycota	fungi
Martensiomyces pterosporus	5	5	zoopagomycota	fungi
Kickxella alabastrina	6	6	zoopagomycota	fungi
Linderina pennispora	6	6	zoopagomycota	fungi
Coemansia mojavensis	6	6	zoopagomycota	fungi
Umbelopsis isabellina	6	6	mucoromycota	fungi
Lunasporangiospora selenospora	6	6	mucoromycota	fungi
Linnemannia elongata	10	10	mucoromycota	fungi
Dissophora decumbens	8	8	mucoromycota	fungi
Gamsiella multidivaricata	8	8	mucoromycota	fungi
Actinomortierella wolfii	7	7	mucoromycota	fungi
Lobosporangium transversale	8	8	mucoromycota	fungi
Glomus cerebriforme	7	7	glomeromycetes	fungi
Rhizophagus clarus	6	6	glomeromycetes	fungi
Gigaspora margarita	7	10	glomeromycetes	fungi
Bifiguratus adelaidae	6	6	other fungi	fungi
Jimgerdemannia flammicorona	6	7	other fungi	fungi
Endogone sp. FLAS-F59071	4	4	other fungi	fungi
Pilobolus umbonatus	5	5	other fungi	fungi
Chaetocladium brefeldii	6	6	other fungi	fungi
Mycotypha africana	6	6	other fungi	fungi
Radiomyces spectabilis	5	5	other fungi	fungi
Lichtheimia ramosa	8	8	other fungi	fungi
Fennellomyces linderi	9	9	other fungi	fungi
Circinella umbellata	9	9	other fungi	fungi
Zychaea mexicana	8	8	other fungi	fungi
Dichotomocladium robustum	8	8	other fungi	fungi
Phascolomyces articulosus	9	9	other fungi	fungi
Lentamyces zychae	6	6	other fungi	fungi
Helicostylum pulchrum	6	6	other fungi	fungi
Thamnidium elegans	6	6	other fungi	fungi
Kirkomyces cordense	6	6	other fungi	fungi
Ellisomyces anomalus	7	7	other fungi	fungi
Cokeromyces recurvatus	6	6	other fungi	fungi
Pilaira anomala	6	6	other fungi	fungi
Benjaminiella poitrasii	6	6	other fungi	fungi
Parasitella parasitica	6	6	other fungi	fungi
Amylomyces rouxii	7	7	other fungi	fungi

Mucor mucedo	5	5	other fungi	fungi
Syncephalastrum racemosum	6	6	other fungi	fungi
Gilbertella persicaria var. persicaria	6	6	other fungi	fungi
Choanephora cucurbitarum	6	6	other fungi	fungi
Blakeslea trispora	6	6	other fungi	fungi
Sporodiniella umbellata	6	6	other fungi	fungi
Rhizopus stolonifer	8	8	other fungi	fungi
Spinellus fusiger	7	7	other fungi	fungi
Phycomyces blakesleeanus	7	7	other fungi	fungi
Hesseltinella vesiculosa	4	4	other fungi	fungi
Halteromyces radiatus	5	5	other fungi	fungi
Gongronella butleri	5	5	other fungi	fungi
Chlamydoabsidia padenii	6	6	other fungi	fungi
Cunninghamella echinulata	5	5	other fungi	fungi
Absidia caerulea	6	6	other fungi	fungi
Gonapodya prolifera JEL478	8	8	chytrids	fungi
Hyaloraphidium curvatum	8	8	chytrids	fungi
Anaeromyces robustus	5	5	chytrids	fungi
Neocallimastix californiae	7	7	chytrids	fungi
Piromyces finnis	6	6	chytrids	fungi
Caulochytrium protostelioides	4	4	chytrids	fungi
Blyttiomyces helicus	5	5	chytrids	fungi
Batrachochytrium salamandrivorans	5	10	chytrids	fungi
Globomyces pollinis-pini	5	5	chytrids	fungi
Gorgonomyces haynaldii	4	4	chytrids	fungi
Synchytrium endobioticum	5	5	chytrids	fungi
Polychytrium aggregatum	4	4	chytrids	fungi
Cladochytrium replicatum	7	7	chytrids	fungi
Spizellomyces sp. 'palustris'	7	7	chytrids	fungi
Gaertneriomyces semiglobifer	5	5	chytrids	fungi
Fimicolochytrium jonesii	6	6	chytrids	fungi
Powellomyces hirtus	7	7	chytrids	fungi
Geranomyces variabilis	7	7	chytrids	fungi
Zopfochytrium polystomum	9	9	chytrids	fungi
Phlyctochytrium arcticum	5	5	chytrids	fungi
Chytridium lagenaria	9	9	chytrids	fungi
Rhizoclosmatium globosum	7	7	chytrids	fungi
Obelidium mucronatum	5	5	chytrids	fungi
Entophlyctis luteolus	5	5	chytrids	fungi
Chytriomyces sp.	3	3	chytrids	fungi
Chytriomyces hyalinus	7	7	chytrids	fungi
Albugo laibachii Nc14	4	4	oomycetes	oomycetes
Phytopythium vexans DAOM BR484	7	7	oomycetes	oomycetes
Pythium arrhenomanes ATCC 12531	9	9	oomycetes	oomycetes

Pythium aphanidermatum DAOM BR444	9	9	oomycetes	oomycetes
Globisporangium ultimum DAOM BR144	9	9	oomycetes	oomycetes
Achlya hypogyna	10	10	oomycetes	oomycetes
Saprolegnia parasitica CBS 223.65	15	15	oomycetes	oomycetes
Aphanomyces astaci	9	18	oomycetes	oomycetes
Thraustotheca clavata	14	14	oomycetes	oomycetes
Nothophytophthora sp. Chile5	18	18	oomycetes	oomycetes
Hyaloperonospora arabidopsidis Emoy2	5	5	oomycetes	oomycetes
Peronospora effusa	6	6	oomycetes	oomycetes
Phytophthora lateralis MPF4	8	8	oomycetes	oomycetes
Phytophthora capsici LT1534	8	8	oomycetes	oomycetes
Phytophthora palmivora var. palmivora	9	9	oomycetes	oomycetes
Phytophthora kernoviae	8	8	oomycetes	oomycetes
Phytophthora ramorum	11	11	oomycetes	oomycetes
Phytophthora sojae	7	7	oomycetes	oomycetes
Phytophthora cactorum	9	9	oomycetes	oomycetes
Phytophthora megakarya	3	3	oomycetes	oomycetes
Phytophthora parasitica	9	14	oomycetes	oomycetes
Phytophthora nicotianae	8	8	oomycetes	oomycetes
Phytophthora infestans	8	8	oomycetes	oomycetes
Plasmopara halstedii	4	4	oomycetes	oomycetes
Amoebophrya	5	5	dinoflagellates	dinoflagellates
Fugacium kawagutii	6	6	dinoflagellates	dinoflagellates
Cladocopium goreaui	12	12	dinoflagellates	dinoflagellates
Breviolum minutum	10	10	dinoflagellates	dinoflagellates
Symbiodinium microadriaticum	7	7	dinoflagellates	dinoflagellates
Chroomonas mesostigmatica CCMP1168	1	1	cryptomonads	cryptomonads
Guillardia theta CCMP2712	13	13	cryptomonads	cryptomonads
Cryptophyta sp. CCMP2293	8	8	cryptomonads	cryptomonads
Cryptomonas paramecium	1	1	cryptomonads	cryptomonads
Saccharina japonica	13	13	brown algae	brown algae
Undaria pinnatifida	11	11	brown algae	brown algae
Nemacystus decipiens	16	16	brown algae	brown algae
Cladosiphon okamuranus	15	15	brown algae	brown algae
Ectocarpus siliculosus	11	11	brown algae	brown algae
Galdieria sulphuraria	3	3	red algae	red algae
Cyanidioschyzon merolae	4	4	red algae	red algae
Porphyridium purpureum	5	5	red algae	red algae
Neopyropia yezoensis	4	4	red algae	red algae
Porphyra umbilicalis	2	2	red algae	red algae
Gracilariopsis chorda	6	6	red algae	red algae
Chondrus crispus	4	4	red algae	red algae
Cyanophora paradoxa	10	10	other eukaryotes	other eukaryotes

Prasinoderma coloniale	10	10	other green plants	green plants
Picocystis sp. ML	1	1	green algae	green plants
Chloropicon primus	7	7	green algae	green plants
Ulva mutabilis	9	9	green algae	green plants
Caulerpa lentillifera	9	9	green algae	green plants
Micromonas commoda	7	7	green algae	green plants
Bathycoccus prasinos RCC1105	6	6	green algae	green plants
Ostreococcus tauri	10	10	green algae	green plants
Tetraselmis striata	17	17	green algae	green plants
Pedinomonas minor	8	8	green algae	green plants
Picochlorum sp. 'soloecismus'	8	8	green algae	green plants
Coccomyxa subellipsoidea C-169	12	12	green algae	green plants
Trebouxia sp. A1-2	9	9	green algae	green plants
Asterochloris glomerata	9	9	green algae	green plants
Symbiochloris reticulata	9	9	green algae	green plants
Botryococcus braunii	11	16	green algae	green plants
Micractinium conductrix	12	12	green algae	green plants
Chlorella sorokiniana	13	13	green algae	green plants
Auxenochlorella protothecoides	6	6	green algae	green plants
Raphidocelis subcapitata	14	14	green algae	green plants
Monoraphidium minutum	13	13	green algae	green plants
Chromochloris zofingiensis	14	14	green algae	green plants
Scenedesmus sp. NREL 46B-D3	15	15	green algae	green plants
Desmodesmus armatus	14	14	green algae	green plants
Flechtneria rotunda	14	14	green algae	green plants
Enallax costatus	17	17	green algae	green plants
Tetradesmus obliquus	13	13	green algae	green plants
Tetrabaena socialis	11	11	green algae	green plants
Edaphochlamys debaryana	16	16	green algae	green plants
Gonium pectorale	13	13	green algae	green plants
Volvox carteri	14	15	green algae	green plants
Microglena sp. YARC	11	11	green algae	green plants
Chlamydomonas	14	14	green algae	green plants
Dunaliella salina	12	13	green algae	green plants
Klebsormidium nitens	11	11	uniseriate?filamentous algae	green plants
Chara braunii	5	5	charophyte algae	green plants
Sphagnum fallax	11	20	moss	green plants
Ceratopteris richardii	11	44	ferns	green plants
Mesostigma viride	5	5	charophyte algae	green plants
Spirogloea muscicola	24	24	green algae	green plants
Mesotaenium endlicherianum	7	7	green algae	green plants
Zygnema circumcarinatum	9	9	green algae	green plants
Selaginella moellendorffii	11	11	moss	green plants
Diphasiastrum complanatum	24	75	moss	green plants
Thuja plicata	10	30	moss	green plants

Ceratodon purpureus	9	33	moss	green plants
Physcomitrium patens	11	49	moss	green plants
Marchantia polymorpha	8	11	moss	green plants
Chlorokybus atmophyticus	10	10	streptophyte algae	green plants
Cinnamomum micranthum f. kanehirae	9	9	flowering plants	green plants
Acorus americanus	11	21	flowering plants	green plants
Actinidia chinensis var. chinensis	11	11	flowering plants	green plants
Vaccinium darrowii	9	9	flowering plants	green plants
Nymphaea colorata	13	37	flowering plants	green plants
Kalanchoe fedtschenkoi	14	27	flowering plants	green plants
Macadamia integrifolia	10	23	flowering plants	green plants
Hydrangea quercifolia	8	15	flowering plants	green plants
Dioscorea alata	12	19	flowering plants	green plants
Coffea canephora	8	8	flowering plants	green plants
Eucalyptus grandis	12	28	flowering plants	green plants
Corymbia citriodora	12	18	flowering plants	green plants
Vitis vinifera	11	34	flowering plants	green plants
Spirodela polyrhiza	8	8	flowering plants	green plants
Zostera marina	10	10	flowering plants	green plants
Amborella trichopoda	9	9	flowering plants	green plants
Asparagus officinalis	8	8	flowering plants	green plants
Zingiber officinale	25	62	flowering plants	green plants
Musa acuminata	14	14	flowering plants	green plants
Joinvillea ascendens	11	14	flowering plants	green plants
Ananas comosus	11	11	flowering plants	green plants
Oropetium thomaeum	12	12	flowering plants	green plants
Digitaria exilis	27	27	flowering plants	green plants
Urochloa fusca	13	36	flowering plants	green plants
Aegilops tauschii subsp. strangulata	11	164	flowering plants	green plants
Paspalum vaginatum	12	24	flowering plants	green plants
Echinochloa crus-galli	39	39	flowering plants	green plants
Thinopyrum intermedium	42	75	flowering plants	green plants
Leersia perrieri	13	14	flowering plants	green plants
Miscanthus sinensis	23	38	flowering plants	green plants
Saccharum spontaneum	30	30	flowering plants	green plants
Chasmanthium laxum	12	14	flowering plants	green plants
Panicum virgatum	27	55	flowering plants	green plants
Pharus latifolius	11	21	flowering plants	green plants
Eragrostis tef	31	31	flowering plants	green plants
Eragrostis curvula	25	25	flowering plants	green plants
Brachypodium distachyon	11	45	flowering plants	green plants
Zea mays	15	34	flowering plants	green plants
Triticum aestivum	36	215	flowering plants	green plants
Sorghum bicolor	12	14	flowering plants	green plants
Setaria viridis	13	34	flowering plants	green plants

Secale cereale	12	12	flowering plants	green plants
Oryza barthii	14	19	flowering plants	green plants
Oryza sativa	15	23	flowering plants	green plants
Oryza longistaminata	14	14	flowering plants	green plants
Lolium perenne	12	12	flowering plants	green plants
Hordeum vulgare	15	170	flowering plants	green plants
Eleusine coracana	23	23	flowering plants	green plants
Mikania micrantha	11	11	flowering plants	green plants
Cynara cardunculus var. scolymus	8	8	flowering plants	green plants
Cichorium endivia	14	14	flowering plants	green plants
Cichorium intybus	9	9	flowering plants	green plants
Lactuca sativa	15	25	flowering plants	green plants
Helianthus annuus	14	14	flowering plants	green plants
Ambrosia artemisiifolia	13	13	flowering plants	green plants
Lindenbergia philippensis	11	26	flowering plants	green plants
Sesamum indicum	8	8	flowering plants	green plants
Erythranthe guttata	19	24	flowering plants	green plants
Fraxinus excelsior	15	25	flowering plants	green plants
Olea europaea	10	10	flowering plants	green plants
Ipomoea triloba	12	21	flowering plants	green plants
Nicotiana tabacum	21	33	flowering plants	green plants
Solanum tuberosum	7	15	flowering plants	green plants
Solanum lycopersicum	9	9	flowering plants	green plants
Capsicum annuum	10	10	flowering plants	green plants
Daucus carota	13	13	flowering plants	green plants
Trifolium pratense	11	11	flowering plants	green plants
Vigna unguiculata	11	29	flowering plants	green plants
Pisum sativum	10	19	flowering plants	green plants
Phaseolus vulgaris	11	25	flowering plants	green plants
Medicago truncatula	10	19	flowering plants	green plants
Lupinus angustifolius	13	13	flowering plants	green plants
Lupinus albus	14	14	flowering plants	green plants
Lens ervoides	9	9	flowering plants	green plants
Lens culinaris	9	9	flowering plants	green plants
Glycine max	19	34	flowering plants	green plants
Cicer arietinum	8	8	flowering plants	green plants
Cajanus cajan	10	10	flowering plants	green plants
Arachis hypogaea	18	25	flowering plants	green plants
Linum usitatissimum	15	15	flowering plants	green plants
Ricinus communis	8	8	flowering plants	green plants
Manihot esculenta	8	34	flowering plants	green plants
Hevea brasiliensis	13	34	flowering plants	green plants
Salix purpurea	11	34	flowering plants	green plants
Populus trichocarpa	10	24	flowering plants	green plants
Cucurbita maxima	11	27	flowering plants	green plants
Cucumis sativus	9	14	flowering plants	green plants

Citrullus lanatus	9	9	flowering plants	green plants
Cleome violacea	8	15	flowering plants	green plants
Descurainia sophioides	10	11	flowering plants	green plants
Meniocus linifolius	16	19	flowering plants	green plants
Myagrum perfoliatum	8	8	flowering plants	green plants
Diptychocarpus strictus	10	11	flowering plants	green plants
Euclidium syriacum	8	8	flowering plants	green plants
Malcolmia maritima	12	12	flowering plants	green plants
Cakile maritima	14	15	flowering plants	green plants
Iberis amara	13	13	flowering plants	green plants
Eruca vesicaria	25	26	flowering plants	green plants
Isatis tinctoria	28	30	flowering plants	green plants
Rorippa islandica	11	12	flowering plants	green plants
Caulanthus amplexicaulis	17	20	flowering plants	green plants
Lunaria annua	11	12	flowering plants	green plants
Schrenkiella parvula	9	9	flowering plants	green plants
Camelina sativa	36	39	flowering plants	green plants
Capsella grandiflora	10	10	flowering plants	green plants
Capsella rubella	12	12	flowering plants	green plants
Eutrema salsugineum	8	10	flowering plants	green plants
Stanleya pinnata	8	8	flowering plants	green plants
Boechera stricta	9	10	flowering plants	green plants
Crambe hispanica	12	14	flowering plants	green plants
Arabis alpina	5	6	flowering plants	green plants
Lepidium sativum	14	15	flowering plants	green plants
Thlaspi arvense	9	10	flowering plants	green plants
Sinapis alba	14	14	flowering plants	green plants
Raphanus sativus	17	18	flowering plants	green plants
Brassica oleracea	9	9	flowering plants	green plants
Brassica rapa	16	16	flowering plants	green plants
Brassica napus	30	30	flowering plants	green plants
Arabidopsis halleri	10	11	flowering plants	green plants
Arabidopsis thaliana	10	14	flowering plants	green plants
Carica papaya	15	15	flowering plants	green plants
Corchorus capsularis	8	8	flowering plants	green plants
Theobroma cacao	8	15	flowering plants	green plants
Gossypium hirsutum	19	40	flowering plants	green plants
Portulaca amilis	9	14	flowering plants	green plants
Amaranthus hypochondriacus	8	8	flowering plants	green plants
Chenopodium quinoa	19	19	flowering plants	green plants
Spinacia oleracea	8	14	flowering plants	green plants
Beta vulgaris	8	8	flowering plants	green plants
Juglans regia	26	26	flowering plants	green plants
Carya illinoinensis	10	30	flowering plants	green plants
Betula platyphylla	14	14	flowering plants	green plants
Corylus avellana	10	10	flowering plants	green plants

Castanea dentata	9	26	flowering plants	green plants
Quercus rubra	11	22	flowering plants	green plants
Ziziphus jujuba	8	23	flowering plants	green plants
Rosa chinensis	12	12	flowering plants	green plants
Fragaria vesca	9	18	flowering plants	green plants
Prunus avium	11	11	flowering plants	green plants
Prunus armeniaca	9	9	flowering plants	green plants
Prunus persica	9	23	flowering plants	green plants
Prunus dulcis	9	11	flowering plants	green plants
Malus domestica	16	16	flowering plants	green plants
Ficus carica	7	7	flowering plants	green plants
Aquilegia coerulea	10	19	flowering plants	green plants
Papaver somniferum	21	21	flowering plants	green plants
Anacardium occidentale	13	47	flowering plants	green plants
Pistacia vera	11	11	flowering plants	green plants
Mangifera indica	14	26	flowering plants	green plants
Citrus clementina	9	17	flowering plants	green plants
Citrus sinensis	8	30	flowering plants	green plants
Acidovorax citrulli	2	2	proteobacteria	proteobacteria
Xanthomonas oryzae	1	1	proteobacteria	proteobacteria
Pseudomonas syringae	1	1	proteobacteria	proteobacteria

Table S3. The classification of *HDAC* genes from the 142 selected species.

Tax name	HDAC1	HDAC3	HDAC8	Class I	HDAC4	HDAC6	HDAC12	Class II	HDAC11	Class IV	Total	Subphyla	Species
Rattus norvegicus	3	1	1	**5**	4	3	0	**7**	1	**1**	**13**	chordates	animals
Oryctolagus cuniculus	2	1	1	**4**	3	2	0	**5**	1	**1**	**10**	chordates	animals
Equus caballus	2	1	1	**4**	4	2	0	**6**	1	**1**	**11**	chordates	animals
Bos taurus	2	1	1	**4**	4	2	0	**6**	1	**1**	**11**	chordates	animals
Felis catus	2	1	1	**4**	4	2	0	**6**	1	**1**	**11**	chordates	animals
Homo sapiens	2	1	2	**5**	4	2	0	**6**	1	**1**	**12**	chordates	animals
Erinaceus europaeus	3	1	1	**5**	2	2	0	**4**	1	**1**	**10**	chordates	animals
Phascolarctos cinereus	2	1	1	**4**	4	2	0	**6**	1	**1**	**11**	chordates	animals
Ornithorhynchus anatinus	3	0	1	**4**	2	2	0	**4**	2	**2**	**10**	chordates	animals
Gallus gallus	2	1	1	**4**	4	1	0	**5**	1	**1**	**10**	chordates	animals
Anas platyrhynchos	2	1	1	**4**	3	1	0	**4**	1	**1**	**9**	chordates	animals
Naja naja	2	1	1	**4**	4	2	0	**6**	1	**1**	**11**	chordates	animals
Pelodiscus sinensis	2	1	1	**4**	4	1	0	**5**	1	**1**	**10**	chordates	animals
Chelydra serpentina	2	1	1	**4**	4	2	0	**6**	2	**2**	**12**	chordates	animals
Lithobates catesbeianus	2	1	0	**3**	2	0	0	**2**	1	**1**	**6**	chordates	animals
Danio rerio	1	2	1	**4**	5	2	0	**7**	2	**2**	**13**	chordates	animals
Ephemera danica	1	0	1	**2**	1	1	2	**4**	1	**1**	**7**	insects	animals
Chrysoperla carnea	1	1	0	**2**	1	1	0	**2**	1	**1**	**5**	insects	animals
Frankliniella occidentalis	1	1	1	**3**	1	1	0	**2**	2	**2**	**7**	insects	animals
Pediculus humanus	1	1	1	**3**	1	1	0	**2**	0	**0**	**5**	insects	animals
Zootermopsis nevadensis	1	2	1	**4**	1	1	0	**2**	1	**1**	**7**	insects	animals
Leptopilina heterotoma	2	1	0	**3**	1	1	0	**2**	0	**0**	**5**	insects	animals
Solenopsis invicta	2	1	0	**3**	1	1	0	**2**	0	**0**	**5**	insects	animals
Apis mellifera	1	1	0	**2**	1	1	0	**2**	0	**0**	**4**	insects	animals
Hermetia illucens	1	1	0	**2**	1	1	0	**2**	2	**2**	**6**	insects	animals
Clunio marinus	2	1	0	**3**	1	1	0	**2**	0	**0**	**5**	insects	animals
Drosophila melanogaster	1	1	0	**2**	1	1	0	**2**	1	**1**	**5**	insects	animals

Zeugodacus cucurbitae	1	1	1	**3**	1	1	0	**2**	1	**1**	**6**	insects	animals
Plutella xylostella	1	1	1	**3**	1	1	0	**2**	1	**1**	**6**	insects	animals
Chilo suppressalis	1	1	1	**3**	1	1	0	**2**	1	**1**	**6**	insects	animals
Spodoptera frugiperda	1	1	1	**3**	1	1	0	**2**	1	**1**	**6**	insects	animals
Bombyx mori	1	1	1	**3**	1	1	0	**2**	1	**1**	**6**	insects	animals
Agrilus planipennis	1	1	1	**3**	1	1	0	**2**	1	**1**	**6**	insects	animals
Leptinotarsa decemlineata	1	1	0	**2**	1	1	0	**2**	1	**1**	**5**	insects	animals
Nilaparvata lugens	1	2	0	**3**	1	1	0	**2**	2	**2**	**7**	insects	animals
Bemisia tabaci	2	1	2	**5**	1	1	0	**2**	1	**1**	**8**	insects	animals
Ditylenchus destructor	1	2	1	**4**	1	2	0	**3**	1	**1**	**8**	nematodes	animals
Bursaphelenchus xylophilus	2	1	0	**3**	1	1	0	**2**	1	**1**	**6**	nematodes	animals
Encephalitozoon hellem ATCC 50504	0	0	2	**2**	0	0	0	**0**	0	**0**	**2**	microsporidians	fungi
Ustilago trichophora	4	2	2	**8**	0	2	0	**2**	0	**0**	**10**	basidiomycete fungi	fungi
Moesziomyces aphidis	1	1	1	**3**	0	1	1	**2**	0	**0**	**5**	basidiomycete fungi	fungi
Sporisorium scitamineum	2	1	1	**4**	0	1	1	**2**	0	**0**	**6**	basidiomycete fungi	fungi
Puccinia sorghi	2	1	1	**4**	0	1	1	**2**	0	**0**	**6**	basidiomycete fungi	fungi
Rhizoctonia solani	2	1	1	**4**	0	1	1	**2**	1	**1**	**7**	basidiomycete fungi	fungi
Hericium coralloides	2	1	1	**4**	0	1	2	**3**	0	**0**	**7**	basidiomycete fungi	fungi
Ganoderma sinense ZZ0214-1	7	1	1	**9**	0	1	2	**3**	0	**0**	**12**	basidiomycete fungi	fungi
Moniliophthora roreri	2	1	1	**4**	0	1	1	**2**	0	**0**	**6**	basidiomycete fungi	fungi
Agaricus bisporus	2	1	1	**4**	0	1	1	**2**	0	**0**	**6**	basidiomycete fungi	fungi
Microbotryum intermedium	2	1	1	**4**	0	1	1	**2**	0	**0**	**6**	basidiomycete fungi	fungi
Cryptococcus neoformans var. grubii	3	1	1	**5**	0	1	1	**2**	0	**0**	**7**	basidiomycete fungi	fungi
Tuber magnatum	1	1	0	**2**	0	1	1	**2**	0	**0**	**4**	ascomycete fungi	fungi
Lachnellula willkommii	1	0	0	**1**	0	1	1	**2**	0	**0**	**3**	ascomycete fungi	fungi
Rhynchosporium secalis	1	1	0	**2**	0	1	1	**2**	0	**0**	**4**	ascomycete fungi	fungi
Botrytis cinerea B05.10	1	1	0	**2**	0	1	1	**2**	0	**0**	**4**	ascomycete fungi	fungi
Monilinia fructicola	1	1	0	**2**	0	1	0	**1**	0	**0**	**3**	ascomycete fungi	fungi
Sclerotinia sclerotiorum	1	1	0	**2**	0	1	1	**2**	0	**0**	**4**	ascomycete fungi	fungi

Pyricularia oryzae	1	1	0	**2**	0	1	1	**2**	0	**0**	**4**	ascomycete fungi	fungi
Diaporthe helianthi	1	1	0	**2**	0	1	1	**2**	0	**0**	**4**	ascomycete fungi	fungi
Valsa mali	1	1	0	**2**	0	1	1	**2**	0	**0**	**4**	ascomycete fungi	fungi
Bretziella fagacearum	1	1	0	**2**	0	1	2	**3**	0	**0**	**5**	ascomycete fungi	fungi
Monosporascus cannonballus	1	1	0	**2**	0	1	1	**2**	0	**0**	**4**	ascomycete fungi	fungi
Verticillium dahliae	1	1	0	**2**	0	1	1	**2**	0	**0**	**4**	ascomycete fungi	fungi
Metarhizium anisopliae	1	1	0	**2**	0	1	1	**2**	0	**0**	**4**	ascomycete fungi	fungi
Fusarium fujikuroi	1	1	0	**2**	0	1	1	**2**	0	**0**	**4**	ascomycete fungi	fungi
Histoplasma mississippiense (nom. inval.)	1	1	0	**2**	0	1	1	**2**	0	**0**	**4**	ascomycete fungi	fungi
Aspergillus flavus	2	1	0	**3**	0	1	1	**2**	0	**0**	**5**	ascomycete fungi	fungi
Pseudocercospora musae	1	1	0	**2**	0	1	0	**1**	0	**0**	**3**	ascomycete fungi	fungi
Boeremia exigua	1	1	0	**2**	0	1	1	**2**	0	**0**	**4**	ascomycete fungi	fungi
Venturia inaequalis	1	1	0	**2**	0	1	0	**1**	0	**0**	**3**	ascomycete fungi	fungi
Saccharomyces cerevisiae	1	1	1	**3**	0	1	1	**2**	0	**0**	**5**	ascomycete fungi	fungi
Lobosporangium transversale	3	1	1	**5**	1	1	1	**3**	0	**0**	**8**	mucoromycota	fungi
Rhizophagus clarus	2	1	0	**3**	0	1	1	**2**	1	**1**	**6**	glomeromycetes	fungi
Piromyces finnis	2	1	2	**5**	0	1	0	**1**	0	**0**	**6**	chytrids	fungi
Synchytrium endobioticum	1	1	1	**3**	0	1	1	**2**	0	**0**	**5**	chytrids	fungi
Pythium arrhenomanes ATCC 12531	1	1	1	**3**	0	0	6	**6**	0	**0**	**9**	oomycetes	oomycetes
Pythium aphanidermatum DAOM BR444	1	1	1	**3**	0	0	6	**6**	0	**0**	**9**	oomycetes	oomycetes
Phytophthora sojae	1	1	0	**2**	0	0	5	**5**	0	**0**	**7**	oomycetes	oomycetes
Plasmopara halstedii	1	1	0	**2**	0	0	2	**2**	0	**0**	**4**	oomycetes	oomycetes
Dunaliella salina	2	1	0	**3**	3	0	2	**5**	4	**4**	**12**	green algae	green plants
Ceratopteris richardii	4	1	0	**5**	2	0	1	**3**	3	**3**	**11**	ferns	green plants
Selaginella moellendorffii	2	1	0	**3**	1	1	5	**7**	1	**1**	**11**	moss	green plants
Physcomitrium patens	2	1	0	**3**	2	1	2	**5**	3	**3**	**11**	moss	green plants
Eucalyptus grandis	2	1	0	**3**	1	5	2	**8**	1	**1**	**12**	flowering plants	green plants
Vitis vinifera	3	2	0	**5**	1	1	3	**5**	1	**1**	**11**	flowering plants	green plants

Spirodela polyrhiza	2	1	0	**3**	1	1	2	**4**	1	**1**	**8**	flowering plants	green plants
Amborella trichopoda	3	1	0	**4**	1	1	2	**4**	1	**1**	**9**	flowering plants	green plants
Asparagus officinalis	3	1	0	**4**	1	1	1	**3**	1	**1**	**8**	flowering plants	green plants
Zingiber officinale	9	2	0	**11**	4	2	6	**12**	2	**2**	**25**	flowering plants	green plants
Musa acuminata	7	1	0	**8**	1	1	2	**4**	2	**2**	**14**	flowering plants	green plants
Ananas comosus	4	2	0	**6**	1	1	2	**4**	1	**1**	**11**	flowering plants	green plants
Digitaria exilis	11	2	0	**13**	2	3	5	**10**	4	**4**	**27**	flowering plants	green plants
Oryza sativa	8	1	0	**9**	1	1	3	**5**	1	**1**	**15**	flowering plants	green plants
Mikania micrantha	2	2	0	**4**	1	2	3	**6**	1	**1**	**11**	flowering plants	green plants
Lactuca sativa	2	1	0	**3**	4	5	2	**11**	1	**1**	**15**	flowering plants	green plants
Helianthus annuus	2	2	0	**4**	2	2	5	**9**	1	**1**	**14**	flowering plants	green plants
Ambrosia artemisiifolia	3	3	0	**6**	1	3	2	**6**	1	**1**	**13**	flowering plants	green plants
Sesamum indicum	2	1	0	**3**	1	1	2	**4**	1	**1**	**8**	flowering plants	green plants
Erythranthe guttata	7	1	0	**8**	2	6	2	**10**	1	**1**	**19**	flowering plants	green plants
Olea europaea	6	1	0	**7**	0	1	2	**3**	0	**0**	**10**	flowering plants	green plants
Solanum lycopersicum	3	1	0	**4**	1	1	2	**4**	1	**1**	**9**	flowering plants	green plants
Daucus carota	7	1	0	**8**	1	1	2	**4**	1	**1**	**13**	flowering plants	green plants
Glycine max	10	2	0	**12**	2	2	2	**6**	1	**1**	**19**	flowering plants	green plants
Arachis hypogaea	6	2	0	**8**	2	2	4	**8**	2	**2**	**18**	flowering plants	green plants
Linum usitatissimum	4	2	0	**6**	2	2	4	**8**	1	**1**	**15**	flowering plants	green plants
Manihot esculenta	2	1	0	**3**	1	1	2	**4**	1	**1**	**8**	flowering plants	green plants
Cucumis sativus	3	1	0	**4**	1	1	2	**4**	1	**1**	**9**	flowering plants	green plants
Brassica oleracea	1	1	0	**2**	2	2	2	**6**	1	**1**	**9**	flowering plants	green plants
Arabidopsis thaliana	3	1	0	**4**	1	2	2	**5**	1	**1**	**10**	flowering plants	green plants
Carica papaya	3	2	0	**5**	1	7	1	**9**	1	**1**	**15**	flowering plants	green plants
Theobroma cacao	2	1	0	**3**	1	1	2	**4**	1	**1**	**8**	flowering plants	green plants
Gossypium hirsutum	6	3	0	**9**	2	2	4	**8**	2	**2**	**19**	flowering plants	green plants
Ziziphus jujuba	2	1	0	**3**	1	1	2	**4**	1	**1**	**8**	flowering plants	green plants
Malus domestica	7	2	0	**9**	2	2	2	**6**	1	**1**	**16**	flowering plants	green plants
Pistacia vera	3	1	0	**4**	1	1	3	**5**	2	**2**	**11**	flowering plants	green plants
Mangifera indica	4	1	0	**5**	1	2	4	**7**	2	**2**	**14**	flowering plants	green plants

Citrus sinensis	3	1	0	**4**	0	1	2	**3**	1	**1**	**8**	flowering plants	green plants
Chromera velia CCMP2878	1	4	0	**5**	0	0	3	**3**	1	**1**	**9**	chromeraceae	other eukaryotes
Capsaspora owczarzaki ATCC 30864	1	1	1	**3**	1	0	1	**2**	1	**1**	**6**	filasterea	other eukaryotes
Aureococcus anophagefferens	1	1	0	**2**	0	1	3	**4**	1	**1**	**7**	pelagophytes	other eukaryotes
Tritrichomonas foetus	0	0	5	**5**	0	0	0	**0**	0	**0**	**5**	parabasalia	other eukaryotes
Saccharina japonica	1	2	2	**5**	0	0	6	**6**	2	**2**	**13**	brown algae	brown algae
Plasmodiophora brassicae	1	1	0	**2**	0	0	2	**2**	0	**0**	**4**	endomyxa	other eukaryotes
Labyrinthula sp.	3	1	1	**5**	0	0	1	**1**	0	**0**	**6**	labyrinthulida	bigyra
Cafeteria roenbergensis	1	1	0	**2**	1	0	2	**3**	1	**1**	**6**	bicosoecida	bigyra
Blastocystis hominis	2	1	0	**3**	0	0	1	**1**	0	**0**	**4**	opalinata	bigyra
Aurantiochytrium limacinum ATCC MYA-1381	2	2	1	**5**	0	2	5	**7**	0	**0**	**12**	thraustochytrida	bigyra
Entamoeba histolytica	0	1	0	**1**	0	0	0	**0**	0	**0**	**1**	other eukaryotes	other eukaryotes
Symbiodinium microadriaticum	0	0	0	**0**	0	1	5	**6**	1	**1**	**7**	dinoflagellates	dinoflagellates
Acanthamoeba castellanii str. Neff	2	0	1	**3**	0	0	3	**3**	1	**1**	**7**	other eukaryotes	other eukaryotes
Guillardia theta CCMP2712	1	1	1	**3**	0	3	5	**8**	2	**2**	**13**	cryptomonads	cryptomonads
Nitzschia inconspicua	1	1	0	**2**	0	0	4	**4**	2	**2**	**8**	diatoms	diatoms
Fragilariopsis cylindrus	3	1	0	**4**	0	0	4	**4**	2	**2**	**10**	diatoms	diatoms
Pseudo-nitzschia multistriata	2	1	1	**4**	0	0	2	**2**	2	**2**	**8**	diatoms	diatoms
Thalassiosira oceanica	2	1	1	**4**	0	0	2	**2**	2	**2**	**8**	diatoms	diatoms
Galdieria sulphuraria	1	1	0	**2**	0	0	1	**1**	0	**0**	**3**	red algae	red algae
Theileria orientalis	0	1	0	**1**	0	0	2	**2**	0	**0**	**3**	apicomplexans	apicomplexans
Paramecium tetraurelia	4	5	0	**9**	0	0	2	**2**	2	**2**	**13**	ciliates	ciliates
Phaeocystis globosa	2	1	1	**4**	0	3	9	**12**	2	**2**	**18**	haptophytes	haptophytes
Trypanosoma equiperdum	0	0	2	**2**	0	0	2	**2**	0	**0**	**4**	kinetoplastids	kinetoplastids
Acidovorax citrulli	0	0	0	**0**	0	0	2	**2**	0	**0**	**2**	proteobacteria	proteobacteria
Xanthomonas oryzae	0	0	0	**0**	0	0	1	**1**	0	**0**	**1**	proteobacteria	proteobacteria
Pseudomonas syringae	0	0	0	**0**	0	0	0	**0**	1	**1**	**1**	proteobacteria	proteobacteria

Table S4. Parameter estimates, Ln L values, and LRTs of codon substitution evolutionary analyses of selective patterns for the selected HDACs.

Foreground Branch	Model	p[a]	Parameter estimates<br>(Frequency, f and ω Values)	Ln L	P	BEB[b]
Class II & Class IV	One-ratio model 0 ($\omega_0=\omega_1$)	134	$\omega_0=\omega_1=0.11001$	-142466.38	3.19E-05	NA
	Two-ratio model 2 (ω_0, ω_1)	135	$\omega_0=0.0904145$; $\omega_1=0.116512$	-142463.24		NA
	Model 3 (discrete)	138	-	-138539.86	1.70E-02	NA
	Clade model D (K = 3)	139	site class 0 1 2<br>proportion 0.27406 0.63601 0.08994<br>branch type 0: 0.10274 0.38531 0.02281<br>branch type 1: 0.10274 0.38531 0.02716	-138537.01		NA
	Model A null ($\omega_2=1$)	136	1	-139613.92	1	NA
	Model A ($0<\omega_0<1$)	137	site class 0 1 2a 2b<br>proportion 0.23276 0.23803 0.26164 0.26757<br>background w 0.09658 1.00000 0.09658 1.00000<br>foreground w 0.09658 1.00000 1.00000 1.00000	-139613.92		7*,9**,10**,28**,50**,56**,67*,<br>82**,251**,327**,329**,331**,<br>333**,334*,335**,336**,337**,<br>339**,341*,342**,343**,344,345**,<br>346**,351**,352**,353*,355**,356**,<br>358**,359**,360*,361**,363*,364*,367*,<br>368**,369**,370**,371*,373**,374**,376*,<br>377**,378**,379**,380**,381**,382**,384**,<br>385*,386**,393**,397**,398*,400**

[a] Number of parameters in the ω distribution.

[b] Amino acids from BEB analysis as fixed by PS with posterior probabilities >90% (>95% indicated with one asterisk, >99% indicated with two asterisks) are shown. The numbers corresponding to the positions in HDAC2 (ENSP00000430432.1) of *H. sapiens* were shown in **Figure S14**.
